# *RELN* gene-related drug-resistant epilepsy with periventricular nodular heterotopia treated with radiofrequency thermocoagulation: a case report

**DOI:** 10.3389/fneur.2024.1366776

**Published:** 2024-03-27

**Authors:** Zijian Li, Fuli Wang, Zhidong He, Qi Guo, Jinnan Zhang, Songyan Liu

**Affiliations:** ^1^Department of Neurology, China-Japan Union Hospital of Jilin University, Changchun, China; ^2^Department of Neurosurgery, China-Japan Union Hospital of Jilin University, Changchun, China

**Keywords:** *RELN* gene, periventricular nodular heterotopia, radiofrequency thermocoagulation, stereoelectroencephalography, drug-resistant epilepsy

## Abstract

An increasing number of gene mutations associated with epilepsy have been identified, some linked to gray matter heterotopia—a common cause of drug-resistant epilepsy. Current research suggests that gene mutation-associated epilepsy should not be considered a contraindication for surgery in epilepsy patients. At present, stereoelectroencephalography-guided radiofrequency thermocoagulation is an important method to treat periventricular nodular heterotopia-associated drug-resistant epilepsy. We present a case of drug-resistant epilepsy, accompanied by periventricular nodular heterotopia and a heterozygous mutation of the *RELN* gene, successfully treated with radiofrequency thermocoagulation, resulting in a favorable outcome.

## 1 Introduction

With advancements in molecular genetics, more epilepsy patients undergo genetic testing, leading to the identification of numerous epilepsy-related gene mutations. Traditionally, it was believed that gene mutations contraindicated epilepsy surgery. However, as the understanding of the pathogenic mechanisms and resulting pathophysiological changes in gene mutations has deepened, it has become apparent that surgical treatment is feasible for some patients with mutation-related epilepsy, yielding remarkable therapeutic efficacy (1). While classical resection remains the primary method for treating drug-resistant epilepsy’s epileptogenic foci, it poses risks when targeting deep cortical or functional brain areas. Stereoelectroencephalography-guided radiofrequency thermocoagulation (SEEG-guided RF-TC) offers precise localization, seizure focus or epileptic network destruction, and almost no damage to functional brain areas, making it a viable treatment for various epilepsy types (2, 3). Here, we retrospectively analyze a case of drug-resistant epilepsy characterized by a heterozygous *RELN* gene mutation, periventricular nodular heterotopia (PNH) indicated by cranial magnetic resonance imaging (MRI), unsuccessful seizure control for 6 years with standard anti-seizure medications (ASMs), and subsequent treatment with SEEG-guided RF-TC combined with ASMs. The patient exhibited significant improvement at a 11 months post-coagulation follow-up, as detailed below.

## 2 Case report

The male patient, aged 20, was hospitalized due to a history of seizures spanning 6 years. Initially, 6 years ago, the patient experienced seizures without a discernible trigger. These seizures presented with three primary manifestations. Seizure type 1 (focal aware autonomic seizures) included tightness in the chest, palpitations, and flushing of the cheeks, without any loss of awareness. These symptoms lasted for tens of seconds and occurred several times a day. Seizure type 2 (focal impaired awareness behavior arrest seizures): based on seizure type 1, responsiveness impairment occurs, followed by a blank stare, interruption of ongoing activities, and drop of objects, accompanied by loss of awareness, reddening of the eyes, right hand dystonia, and oral automatisms. These symptoms persisted for dozens of seconds to a minute, followed by a return to a normal state after a brief period of impaired awareness. The seizures occurred approximately 5–6 times a year. Seizure type 3 (focal to bilateral tonic-clonic seizures): based on seizure type 2, appearing head and eye version, followed by tonic and clonic movements of the limbs, foaming at the mouth, loss of awareness, without tongue biting or incontinence. These symptoms lasted tens of seconds and reverted to normal after a brief period of impaired awareness. The seizures occurred approximately once a year. There was no reported history of dystocia, febrile convulsions, encephalitis, or cranio-cerebral trauma, and no family history of epilepsy was reported.

Physical examination: no notable facial features or positive signs were observed during cardiac, pulmonary, abdominal, or neurological examinations. Neurological function assessments revealed a Mini-Mental State Examination (MMSE) score of 29, a Montreal Cognitive Assessment (MoCA) score of 25 (indicative of mild cognitive dysfunction), a Hamilton Depression Scale (HAMD) score of 12 (suggestive of possible anxiety), and a Hamilton Anxiety Scale (HAMA) score of 3 (within normal range).

**Figure 1:**
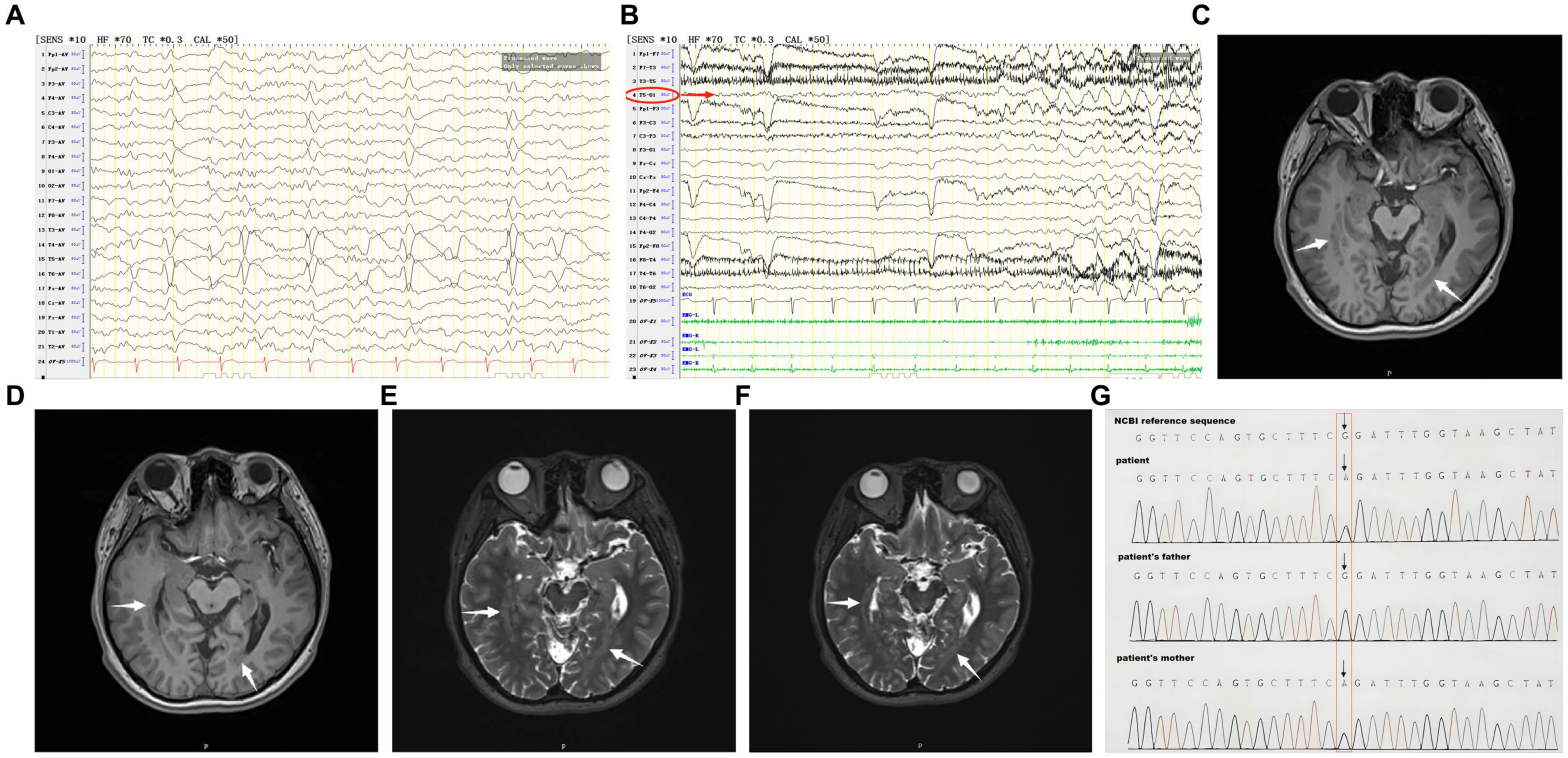
**(A)** The patient’s interictal period EEG. **(B)** The patient’s ictal period EEG. **(C–F)** The patient’s MRI before coagulation. **(G)** Whole-exome sequencing of monogenic disease.

Auxiliary examination: MRI showed heterotopic nodules of grey matter in the bilateral temporal and occipital lobes adjacent to posterior horns of the lateral ventricles, and showed thick bands of heterotopic grey matter extending along the posterior horns of the lateral ventricles; these heterotopic nodules are isointense to grey matter on both T1- and T2-weighted images (Figures 1C–F). PET/MRI revealed unevenly attenuated glucose metabolism in heterotopic nodules. Whole-exome sequencing for monogenic diseases identified a heterozygous missense mutation: *RELN* c.331G>A, amino acid alteration: p.G111R (amino acid 111 was mutated from glycine to arginine). This mutation was present in the patient and a heterozygous mutation of the same allele was found in the patient’s asymptomatic mother, while the father had a wild-type genotype (Figure 1G).

Treatment process: the patient has been taking lamotrigine and valproic acid regularly for approximately 5 years. But up to now, seizure type 1 and seizure type 2 are still not well controlled.

Clinical diagnosis: drug-resistant epilepsy, seizure types: focal aware autonomic seizures, focal impaired awareness behavior arrest seizures, and focal to bilateral tonic-clonic seizures, epilepsy type: focal epilepsy, epilepsy etiology: PNH, mutation in the *RELN* gene, epilepsy comorbidity: anxiety disorder.

**Figure 2:**
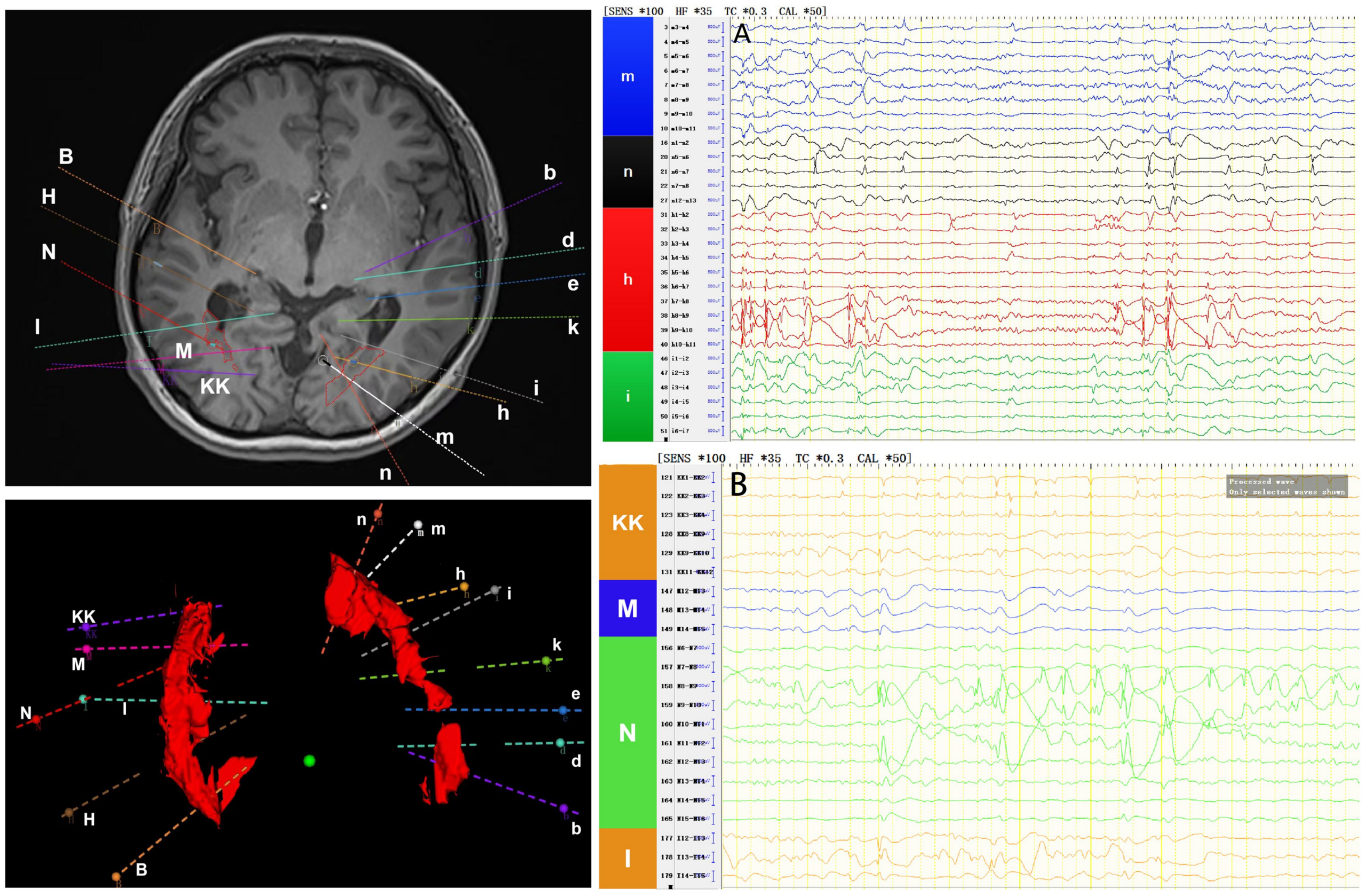
The patient’s interictal period SEEG before coagulation. **(A)** Left electrodes (m/n: occipital lobe-occipital horn nodes; h/i: temporal-occipital junction-occipital horn nodes). **(B)** Right electrodes (KK/M: temporal-occipital junction-occipital horn nodes; N: superior temporal gyrus-atrial nodes; I: middle temporal gyrus posterior-temporal horn nodes).

**Figure 3:**
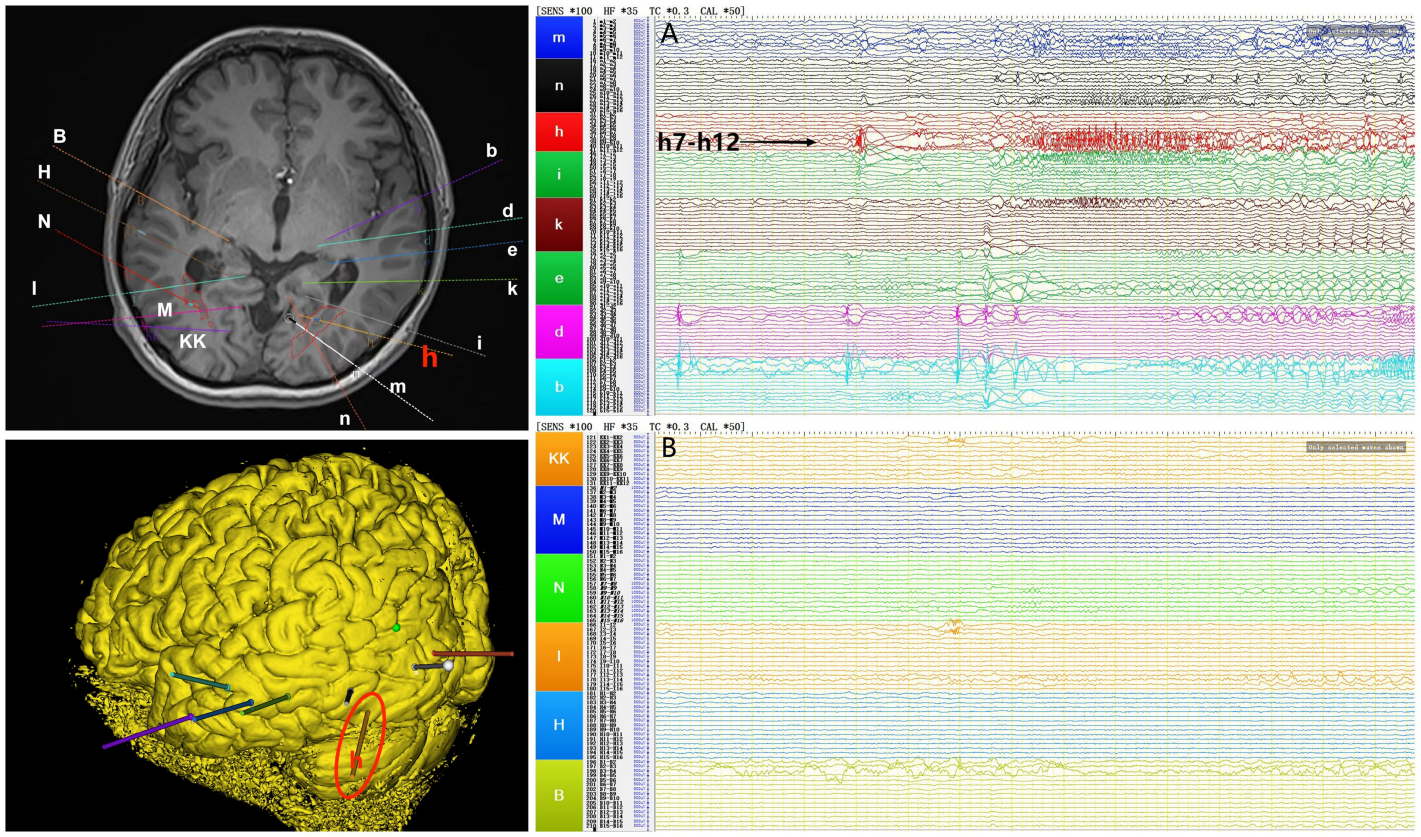
The patient’s ictal period SEEG before coagulation. **(A)** Left electrodes (m/n: occipital lobe-occipital horn nodes; h/i: temporal-occipital junction-occipital horn nodes; k: middle temporal gyrus-atrial nodes; e/d/b: middle temporal gyrus-temporal horn nodes). **(B)** Right electrodes (KK/M: temporal-occipital junction-occipital horn nodes; N: superior temporal gyrus-atrial nodes; I: middle temporal gyrus posterior-temporal horn nodes; H/B: middle temporal gyrus-temporal horn nodes).

Interictal period EEG showed slow spike-wave discharges originated from bilateral temporal lobes (Figure 1A). EEG registered three times seizures during the ictal period, including once focal impaired awareness behavior arrest seizure and twice focal to bilateral tonic-clonic seizures. During the ictal period, EEG registered the epileptiform discharges originating from the left temporal-occipital junction and gradually generalized to the bilateral cerebral hemispheres (Figure 1B). SEEG registered epileptiform discharges mainly originated from heterotopic nodes in the left occipital lobe, temporal-occipital junctions (both left and right), right superior temporal gyrus, and the middle temporal gyrus posterior during the interictal period (Figures 2A,B). Ictal onset was registered from the left temporal-occipital junction heterotopic nodes on SEEG (leads h7–h12; Figures 3A,B). Considering the diffuse distribution of the patient’s heterotopic gray matter nodes in both cerebral hemispheres and the possible involvement of optic radiation by the heterotopic nodes, we chose to perform SEEG-guided RF-TC on the patient, which has almost no damage to brain function. Coagulation procedure: Monopolar coagulations coagulated at least all selected contacts in the left heterotopic nodes. To make the coagulation more adequate, some contacts in the left “core lesion” were additionally coagulated by bipolar coagulations. Because no significant epileptiform discharges were registered in the right hemisphere on SEEG during the ictal period (only slow waves were registered) and epileptiform discharges in the right hemisphere were registered in the interictal period, and in order to prevent the right heterotopic nodes from becoming potential epileptic lesion in the future, monopolar coagulations were used in all of the selected contacts in the right heterotopic nodes. Parameters of RF-TC: 7 W, 30 s, the time interval of repeated coagulation are more than 1 min. Post-coagulation SEEG registered a significant reduction in epileptiform discharges during the interictal period. After coagulation, the patient has been taking lamotrigine and valproic acid regularly.

**Figure 4:**
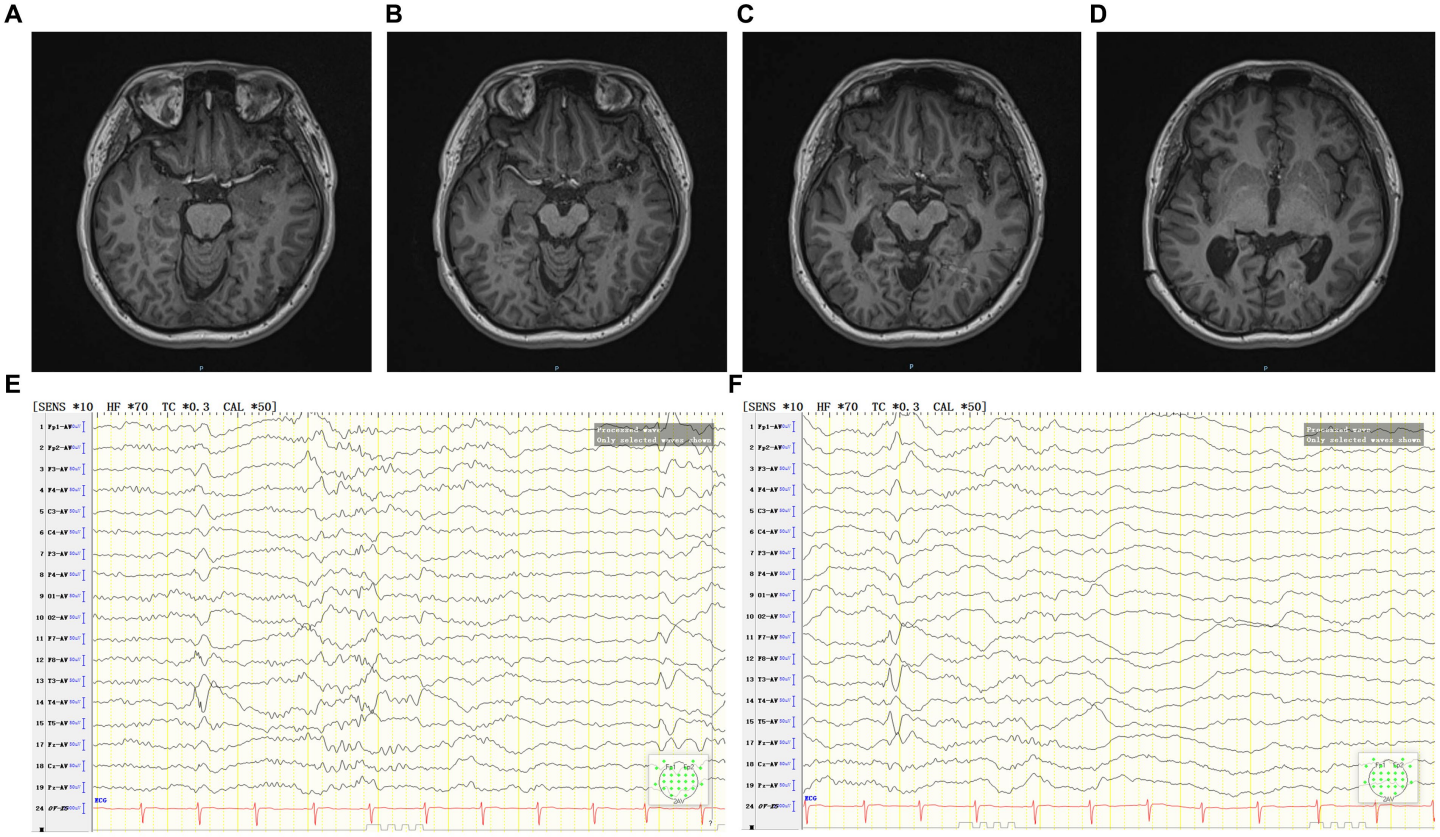
**(A–D)** The patient’s MRI 2 months after coagulation. **(E,F)** The patient’s interictal period EEG 2 months after coagulation.

Follow-up after coagulation (Figures 4A–F): at the 2 months after coagulation, EEG registered occasional epileptiform discharges in bilateral temporal lobes during the interictal period. By the 11 months follow-up, there was only one focal impaired awareness behavior arrest seizure due to stop taking medicine without permission. Presently, the patient continues ASMs treatment, exhibiting a MoCA score of 28, indicating no cognitive dysfunction. Furthermore, there have been no reported complaints of headache, dizziness, vision impairments, or visual field deficits.

## 3 Discussion

This is a case of drug-resistant epilepsy associated with mutations in the *RELN* gene, which, notably, does not contraindicate epilepsy surgery. The mutation in the *RELN* gene presumably caused the PNH, which was found to be responsible for drug-resistant epilepsy. PNH serves as an indication for SEEG-guided RF-TC, which is currently exhibiting effectiveness and absence of cerebral impairment in this patient.

Gray matter heterotopia (GMH) represents a malformation in cortical development (MCD) arising from neuronal migration failure during brain development. GMH categories include PNH, subcortical heterotopia, and subcortical band heterotopias, with PNH being the most prevalent (4, 5). Epilepsy frequently manifests as the main symptom of PNH, and the relationship between PNH and epilepsy is intricate. Abnormal interconnections form between heterotopic nodules and between these nodules and the surrounding cortex, creating epileptic networks, thereby contributing to epilepsy development. PNH commonly results in drug-resistant epilepsy, which underscores the necessity for surgical intervention (6–8).

The human *RELN* gene resides on chromosome 7q22, generating the glycoprotein Reelin (9). Reelin is predominantly synthesized and secreted by Cajal–Retzius (CR) cells in the marginal zone during embryonic and early life stages, with secretion continuing by γ-aminobutyric acidergic neurons and a limited number of CR cells after 18 days of life (9). Various signaling pathways execute downstream signaling post-Reelin binding to cell membrane receptors (9). The receptors of reelin are mainly apolipoprotein E receptor 2 (Apoer2) and very low-density lipoprotein receptor (Vldlr). The binding of reelin to these receptors triggers tyrosine phosphorylation of the protein disabled-1 (Dab1), thus starting a series of intracellular cascade reactions (9, 10). It is found that the reelin signaling pathway mainly interacts with tubulin and actin through Reelin-Dab1-PI3K-AKT, Reelin-Lis1/DCX, and Reelin-cofilin pathways to participate in the processes of neuronal migration, localization, and cortical plate formation, while Reelin-NMDAR pathway is mainly involved in regulating synaptic plasticity and plays an important role in learning and memory (9, 11–13). Aberrations in this pathway correlate closely with diverse psychoneurological disorders such as MCD, epilepsy, schizophrenia, and autism (14). GMH occurrence correlates with mutations in genes linked to cell proliferation, migration, and localization, implicating the involvement of the *RELN* gene (15, 16). Reelin can exert a “stop signal” for migrating neurons (10). Mutations in the *RELN* gene may lead to specific cortical developmental malformations associated with epilepsy (15, 16). For instance, the Reelin-PI3K-AKT-mTOR pathway, an integral part of the Reelin signaling pathway, involves the *RELN* gene as an upstream-related gene of the mTOR pathway (17–19). Studies confirm that mutations in the *AKT* gene disrupting this pathway are closely linked to PNH development (17–19). Rossini et al. (20) conducted a study examining post-surgical specimens from five PNH patients through neuropathology and immunohistochemistry, revealing Reelin-positive cells devoid of typical CR cell characteristics within the heterotopic gray matter nodules. Thus, it is postulated that mutations in the *RELN* gene could disrupt the Reelin pathway, potentially contributing to PNH development. Consequently, the patient’s PNH etiology in this case is believed to correlate with a heterozygous *RELN* gene mutation, consistent with the aforementioned findings. Nevertheless, a paucity of research reports concerning the correlation between the *RELN* gene mutations and PNH necessitates further studies to confirm their relationship.

In recent years, it has been found that the *RELN* gene is one of the primary pathogenic genes of autosomal dominant lateral temporal lobe epilepsy (ADLTE) (21). ADLTE patients are primarily young and middle-aged, with epileptic discharges originating from the lateral temporal lobe and mainly manifested as focal seizures accompanied by auditory aura, which focal to bilateral tonic-clonic may follow (22). Most ADLTE patients are well controlled by medication, and the EEG may be normal in the interictal period, and the MRI may be free of abnormalities (23). However, in this case, the patient had drug-resistant epilepsy caused by PNH, and both EEG and MRI showed positive findings, and the patient is not accompanied by auditory aura, so we do not diagnose this patient as ADLTE. This time, we report a new case of drug-resistant epilepsy associated with the *RELN* gene mutation, which may have implications for further study of the pathophysiology of the *RELN* gene mutation.

The conventional perspective often deems gene mutations as contraindications for epilepsy surgery. Nonetheless, the patient in this case study exhibited a *RELN* gene mutation leading to PNH, resulting in drug-resistant epilepsy. This case presented clear indications for surgery, and over a 11 months follow-up, there was a significant improvement in seizure frequency and severity. Stevelink et al. (1) reported on 30 patients with drug-resistant epilepsy due to mutations in mTOR pathway-related genes. Among these patients, 12 had germline mutations and 18 had somatic mutations. Surgical treatment was undergone by all 30 patients, with 58% of those with germline mutations achieving complete seizure control, 25% showing significant improvement, and 83% of those with somatic mutations achieving complete seizure control. Therefore, despite genetic mutations being associated with MCD in some epilepsy patients, surgical treatment remains viable for patients with limited lesions and well-defined epileptogenic zones, potentially resulting in better efficacy for those with genetic abnormalities and positive imaging findings (1, 24).

The patient’s MoCA score was 25 before coagulation, likely influenced by epilepsy seizures and frequent interictal period discharges. The score improved to 28, which was attributed to seizure control, reduced interictal period discharges, and no neurological damage after SEEG-guided RF-TC. This case suggests that SEEG-guided RF-TC may also be suitable for PNH caused by some gene mutations. Presently, drug-resistant epilepsy linked with PNH is an indication for SEEG-guided RF-TC (25), which is a procedure offering diagnostic and therapeutic roles, showing improved safety and efficiency compared to traditional seizure focus resection (2, 25). SEEG-guided RF-TC shows apparent efficacy in treating certain types of drug-resistant epilepsy and is anticipated to become a primary treatment for PNH-related drug-resistant epilepsy.

## 4 Conclusion

This case’s features hint at a plausible correlation between *RELN* gene mutation and PNH, indicating potential surgical indications for patients with drug-resistant epilepsy associated with such mutation. Clinicians should conduct in-depth analyses of pathophysiological alterations and the clinical significance caused by gene mutations.

## Data availability statement

The original contributions presented in the study are included in the article/Supplementary material, further inquiries can be directed to the corresponding author.

## Ethics statement

The studies involving human participants were reviewed and approved by the Ethics Committee of China-Japan Union Hospital of Jilin University. Written informed consent for participation was not required from the participants or the participants’ legal guardians/next of kin in accordance with the national legislation and institutional requirements. Written informed consent was obtained from the individual for the publication of any potentially identifiable images or data included in this article.

## Author contributions

ZL: Writing – original draft, Data curation, Formal analysis, Investigation. FW: Data curation, Formal analysis, Writing – review & editing. ZH: Investigation, Writing – review & editing. QG: Data curation, Writing – review & editing. JZ: Formal analysis, Writing – review & editing. SL: Supervision, Writing – review & editing.
