# Supplementary figures and images for: RELN gene-related drug-resistant epilepsy with periventricular nodular heterotopia treated with radiofrequency thermocoagulation: a case report
Source: Front Neurol. 2024 Mar 27;15:1366776. doi: 10.3389/fneur.2024.1366776 (PMC11004351; doi:10.3389/fneur.2024.1366776)

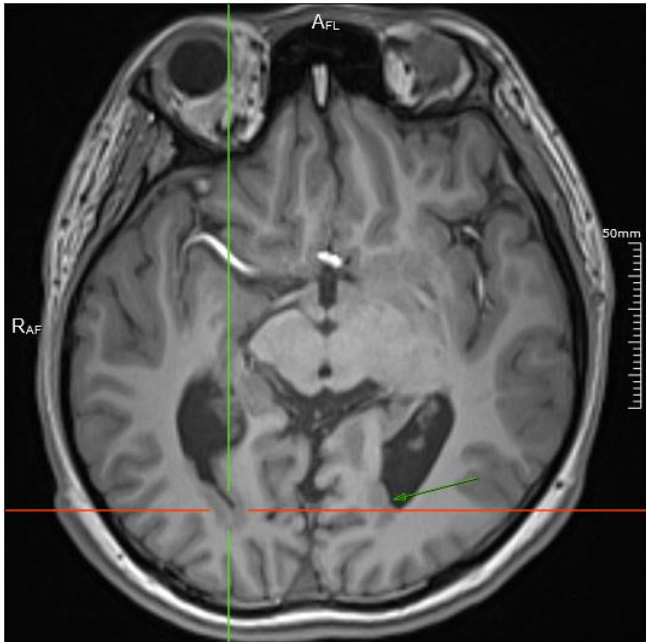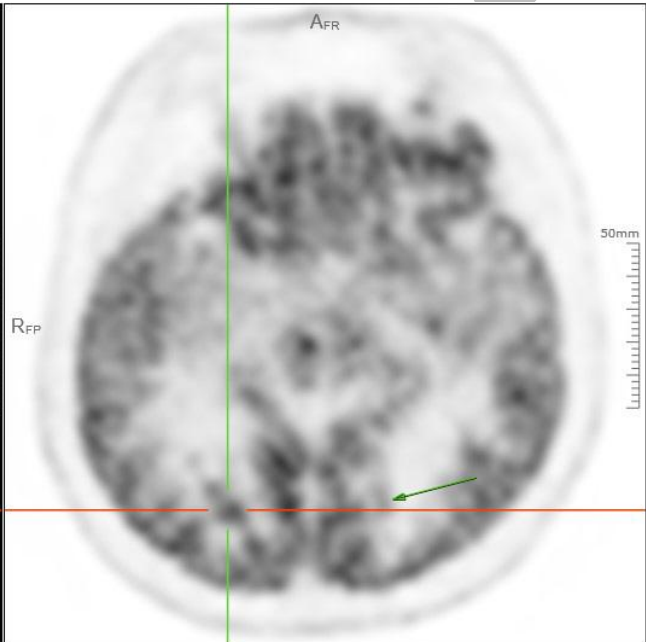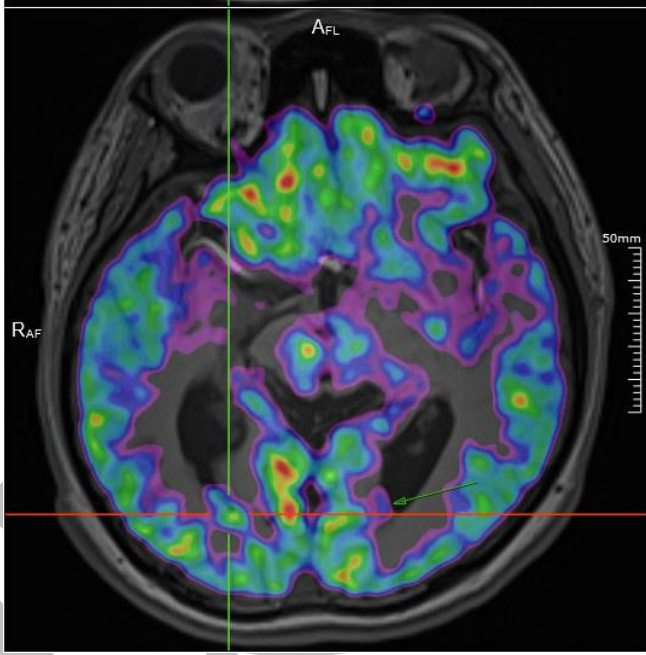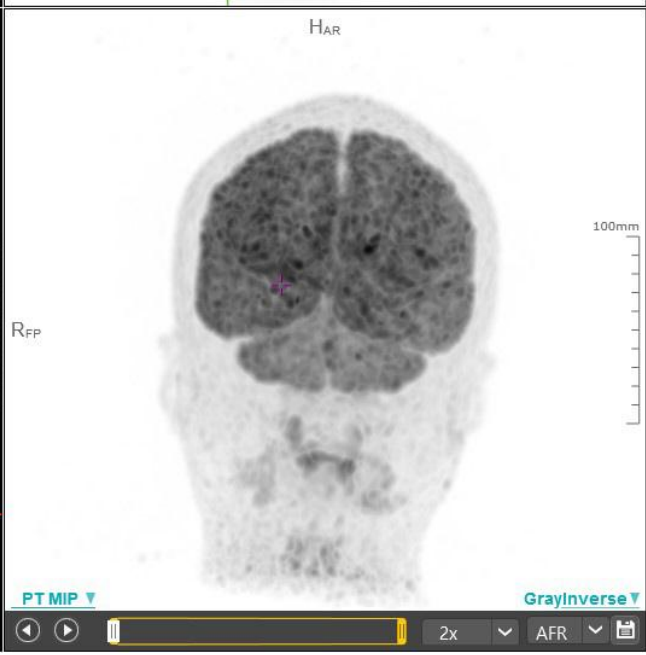

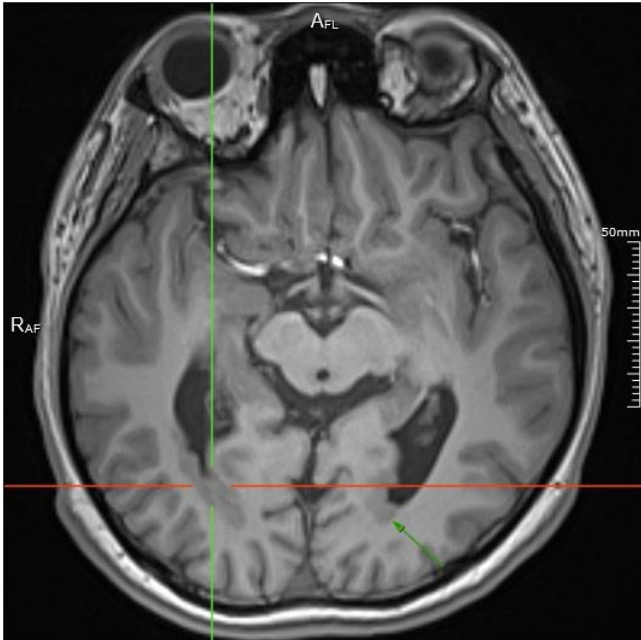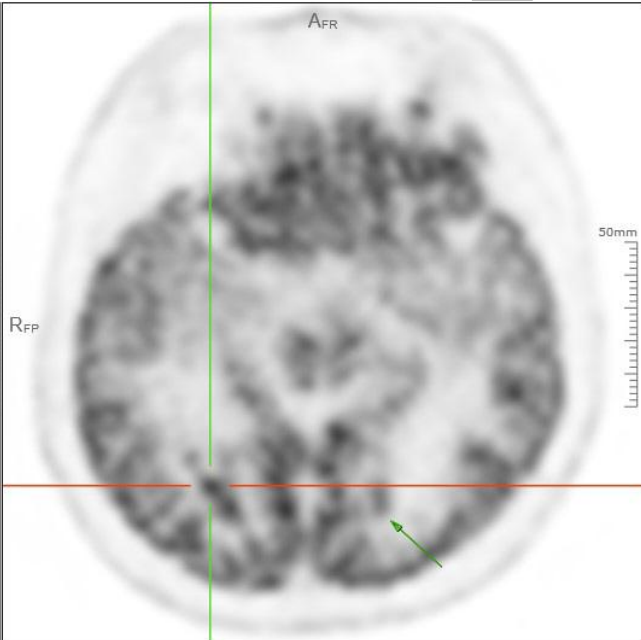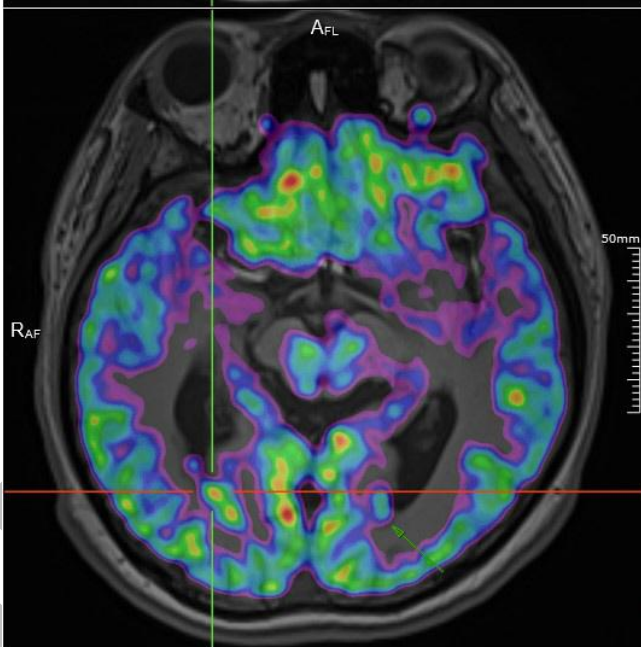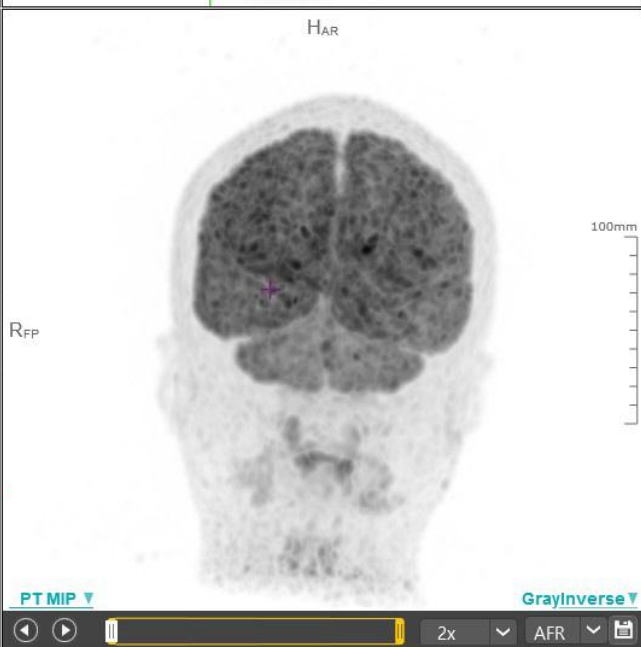

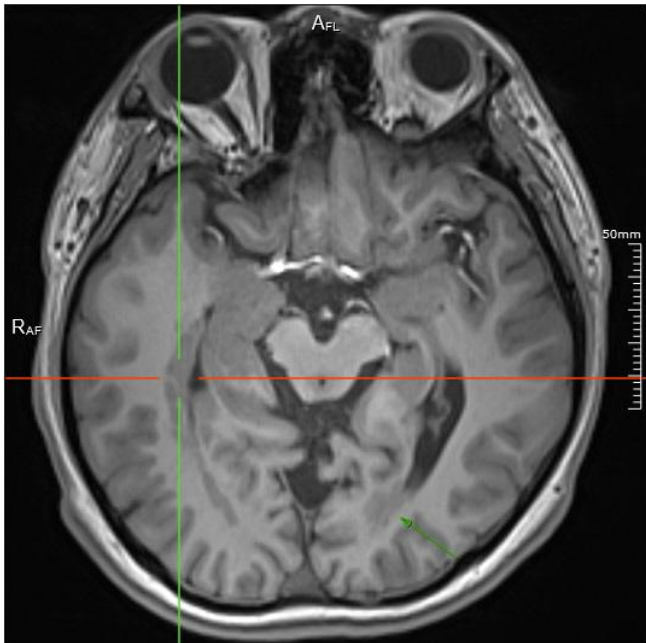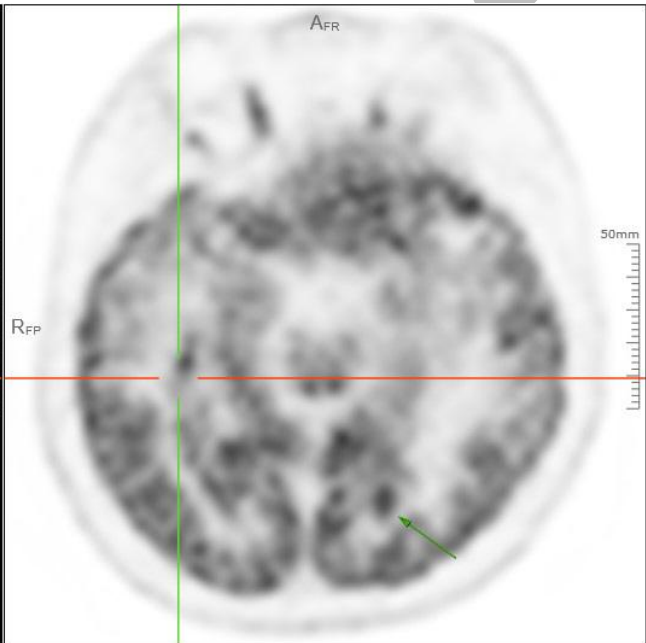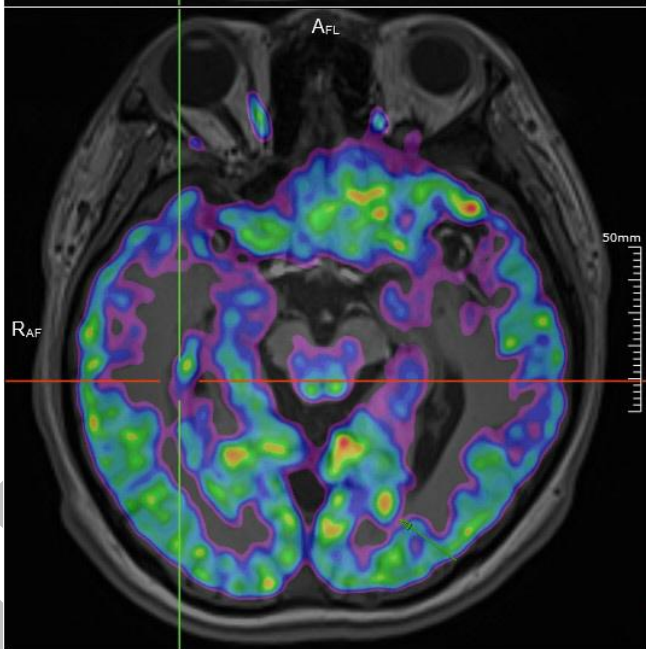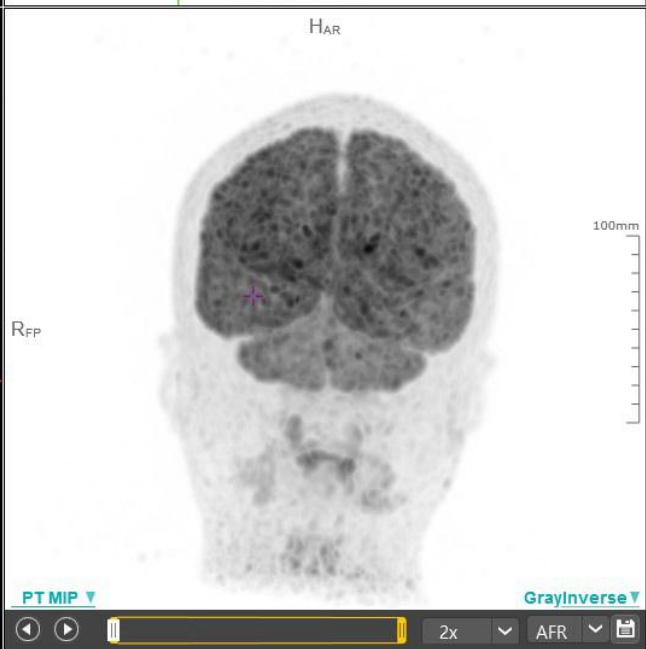

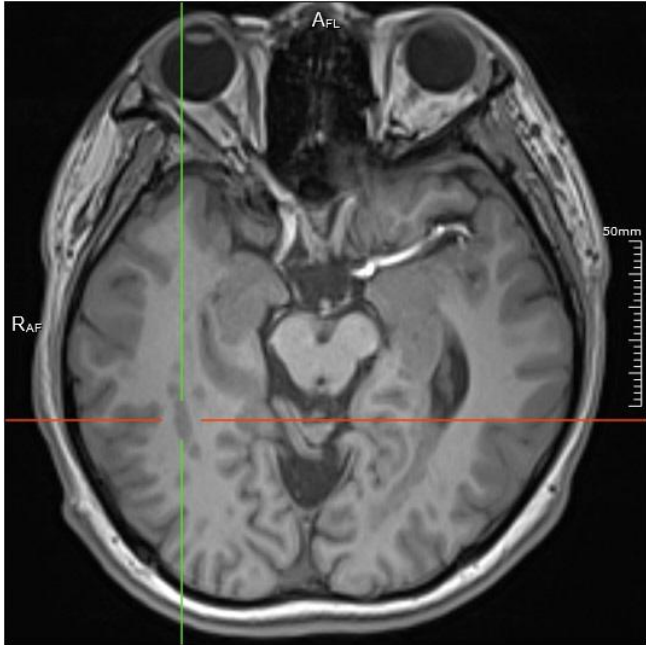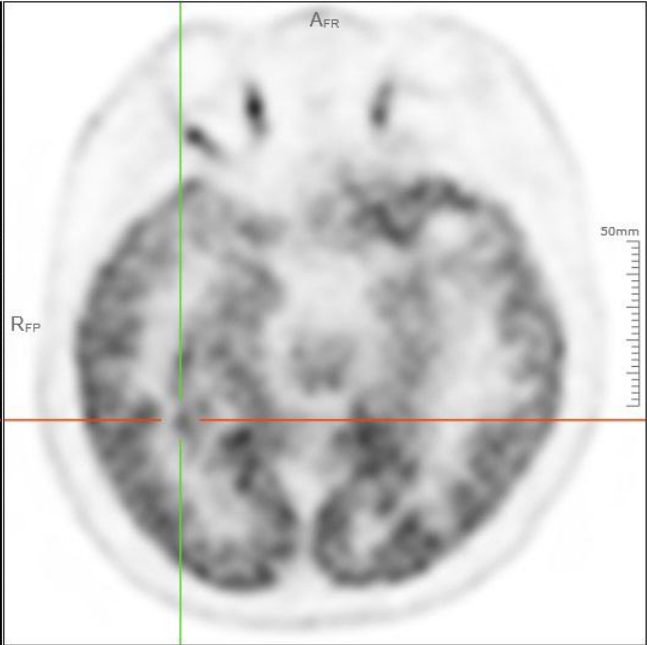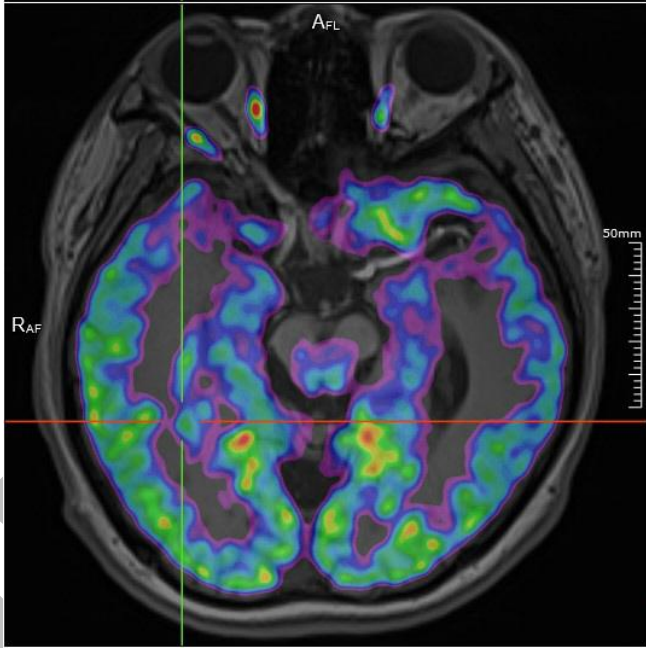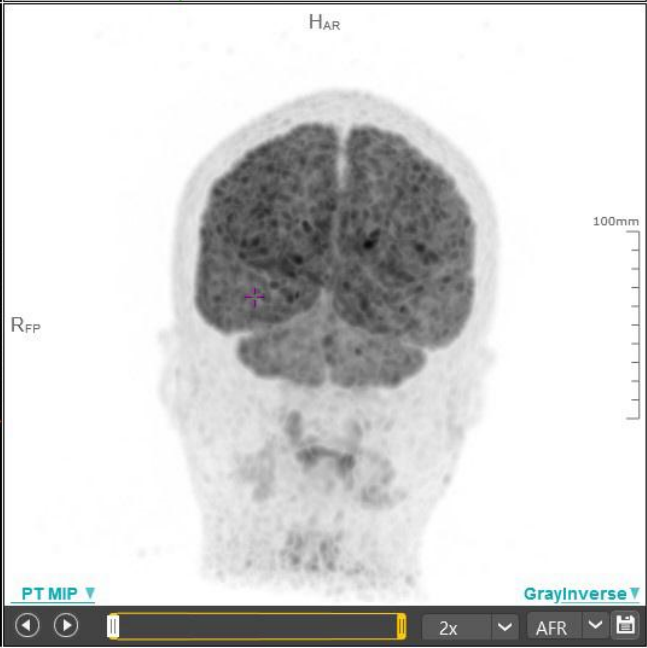

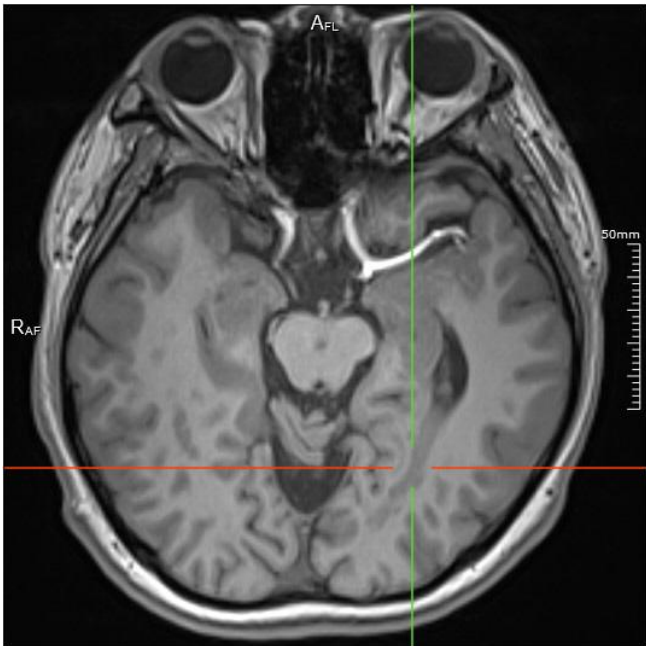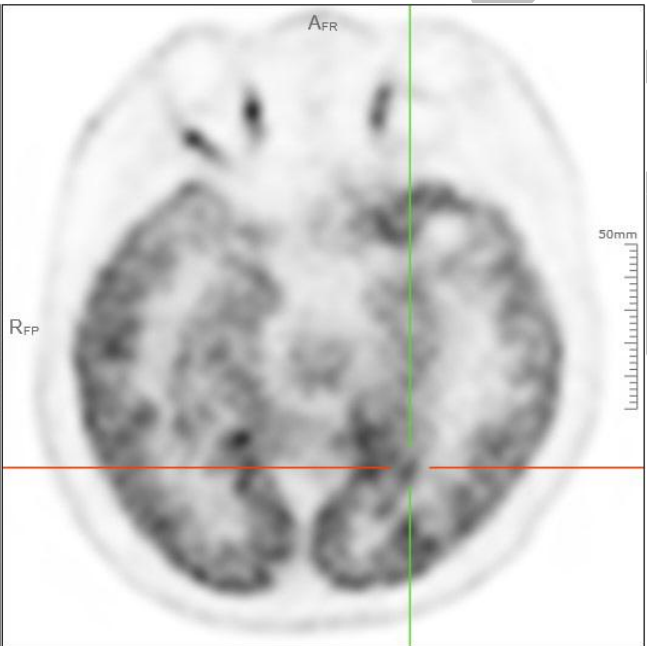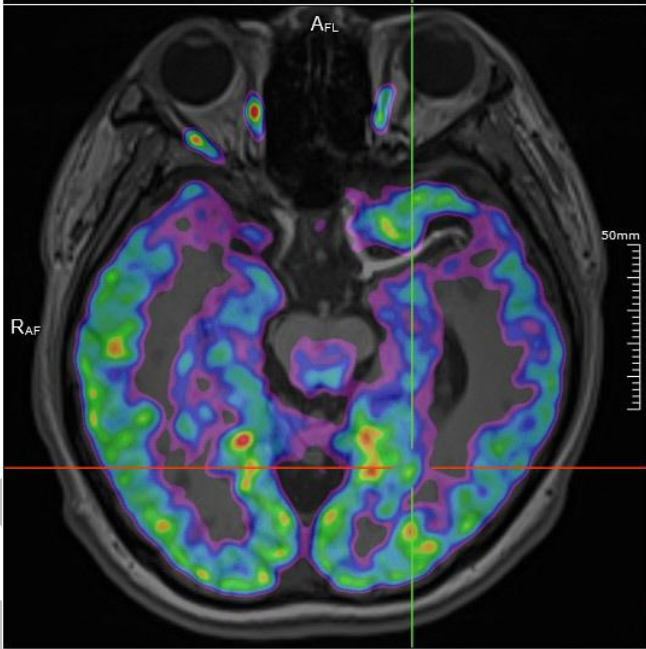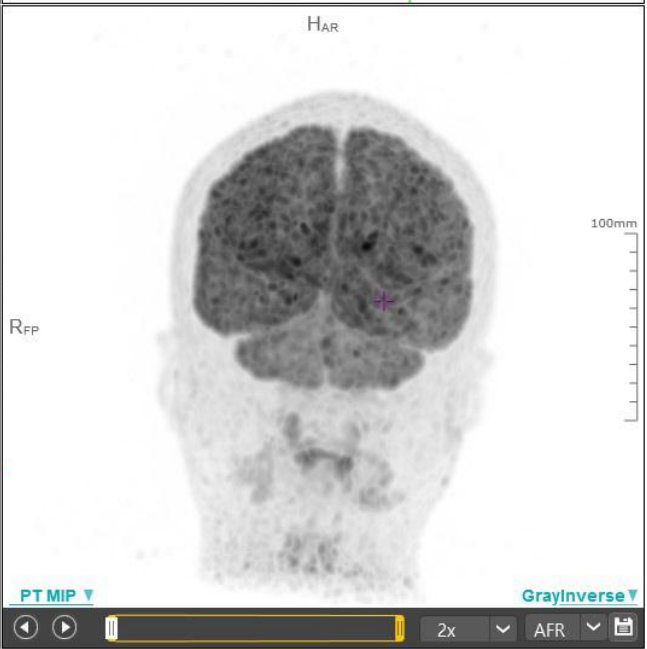

H<sub>AR</sub>

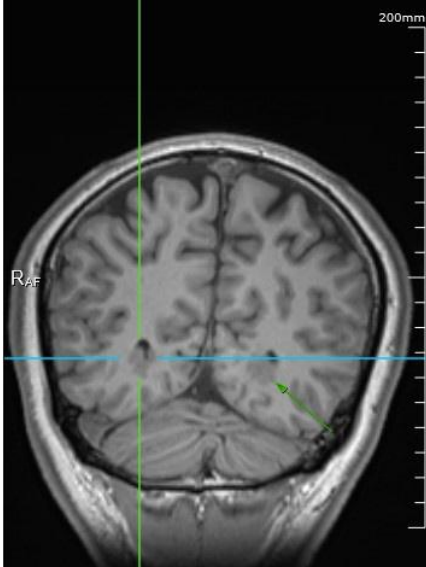

H<sub>AR</sub>

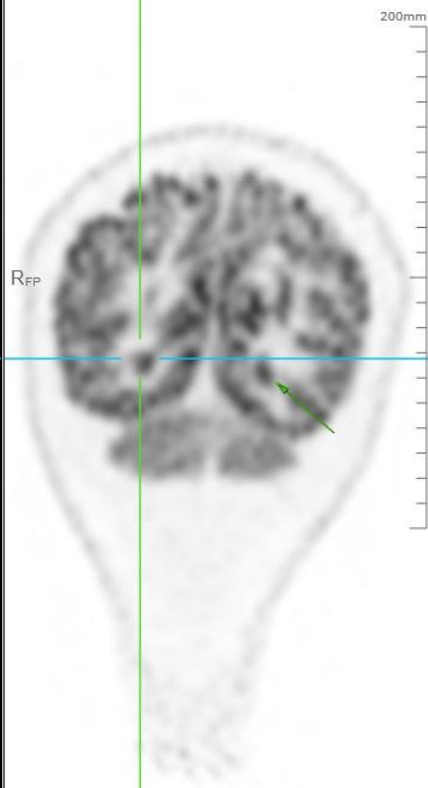

H<sub>AR</sub>

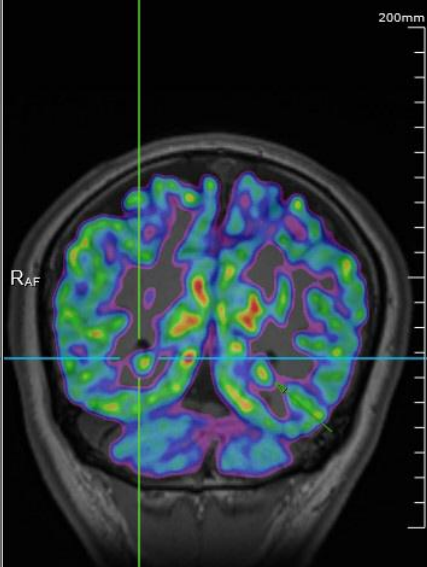

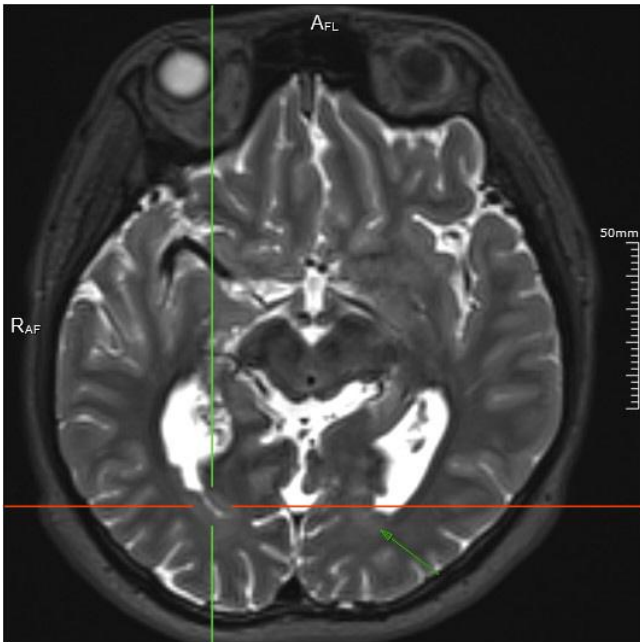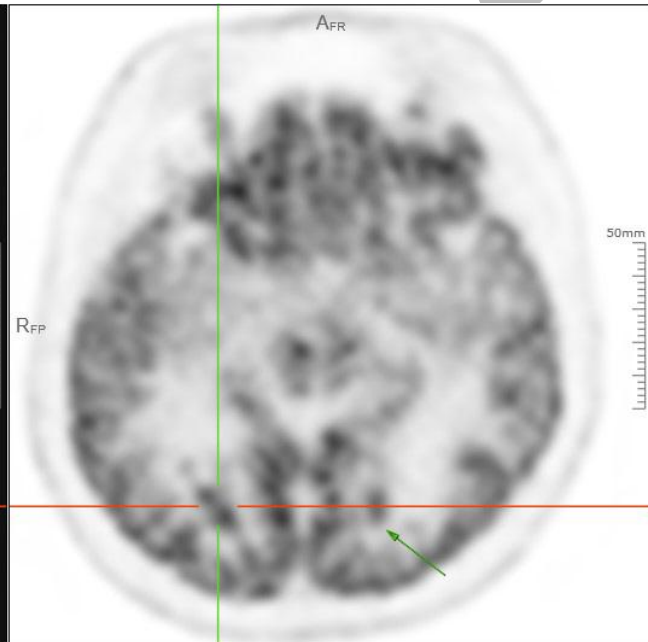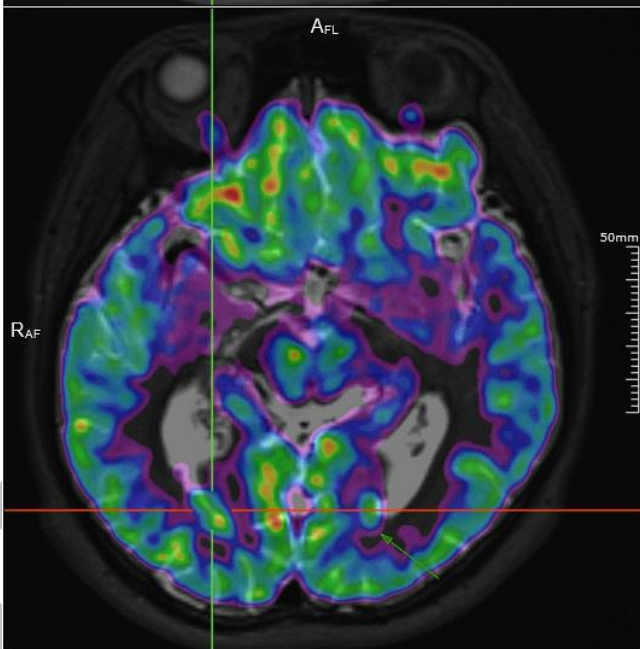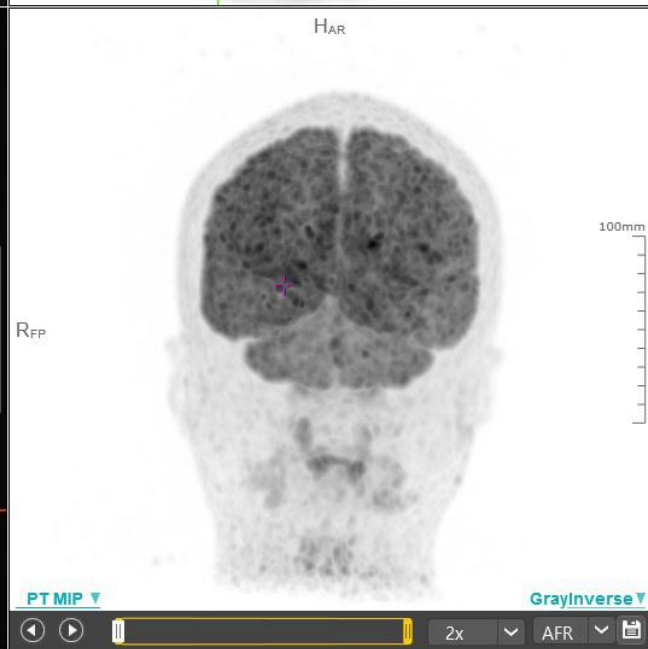

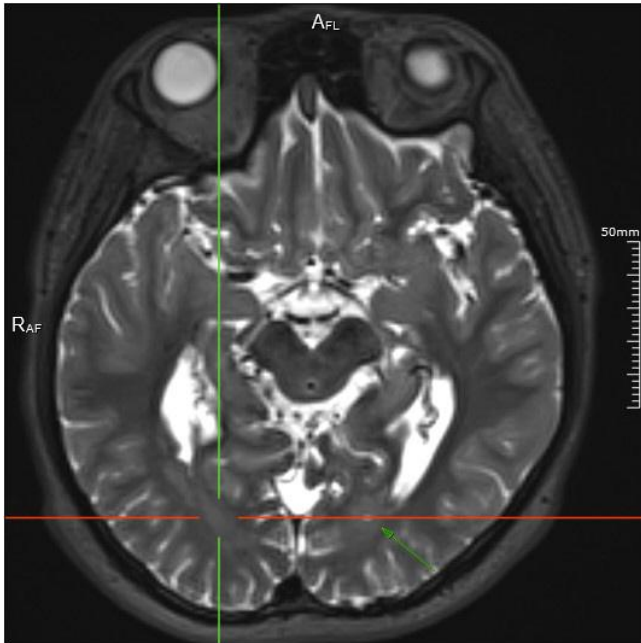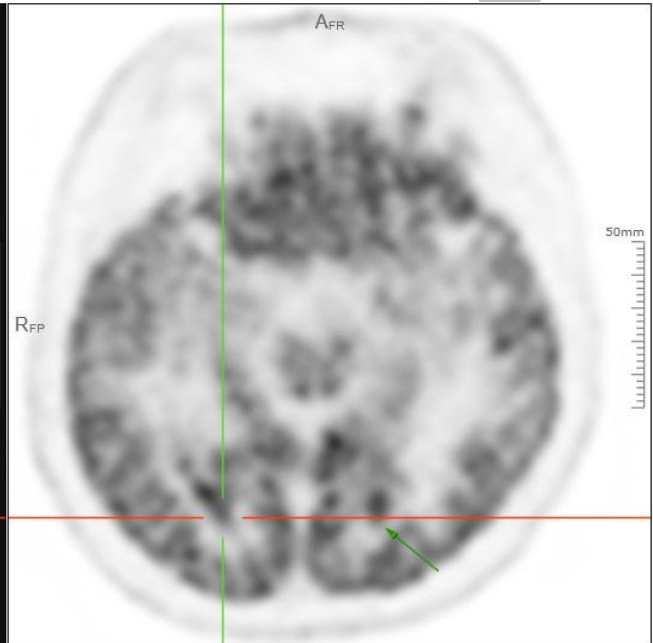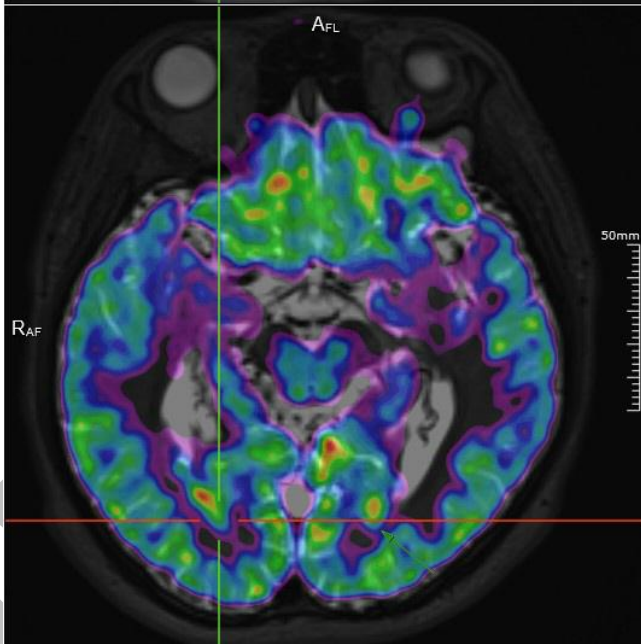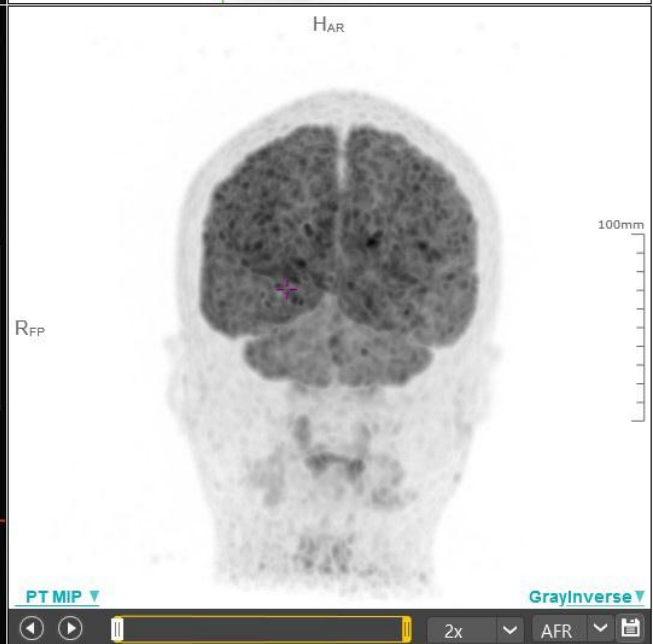

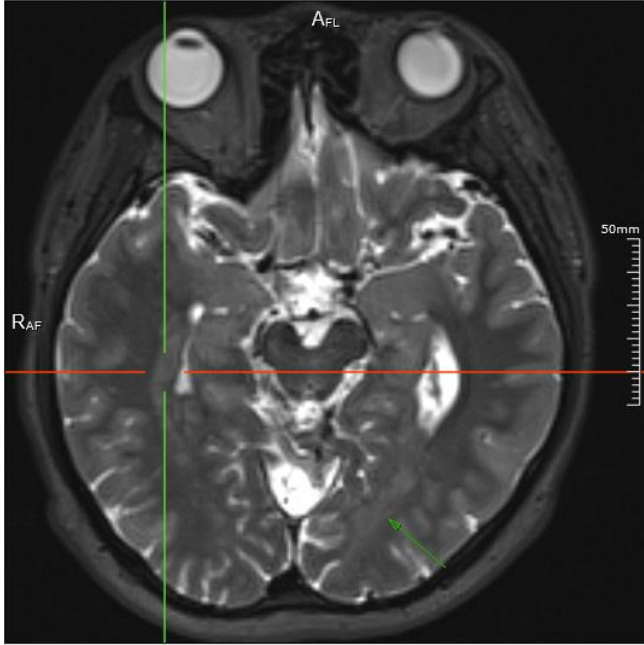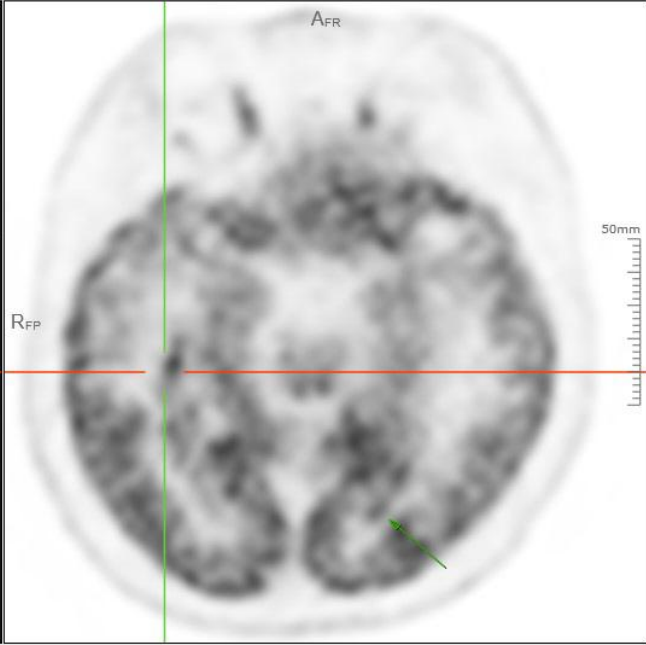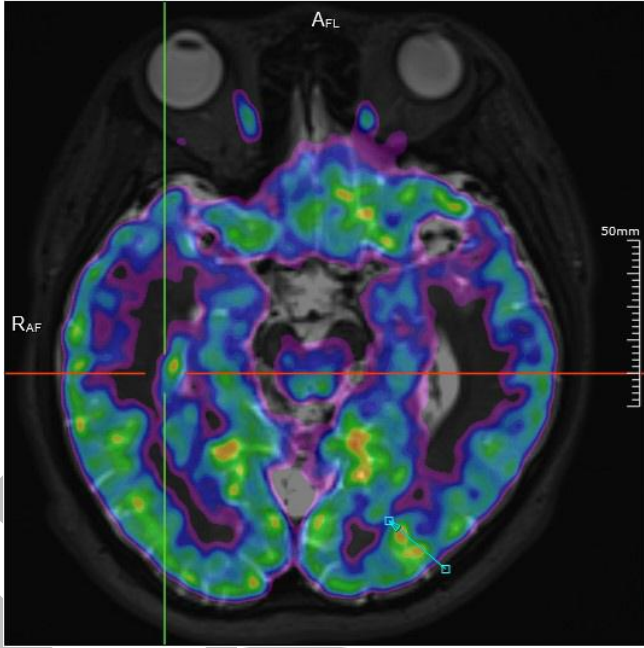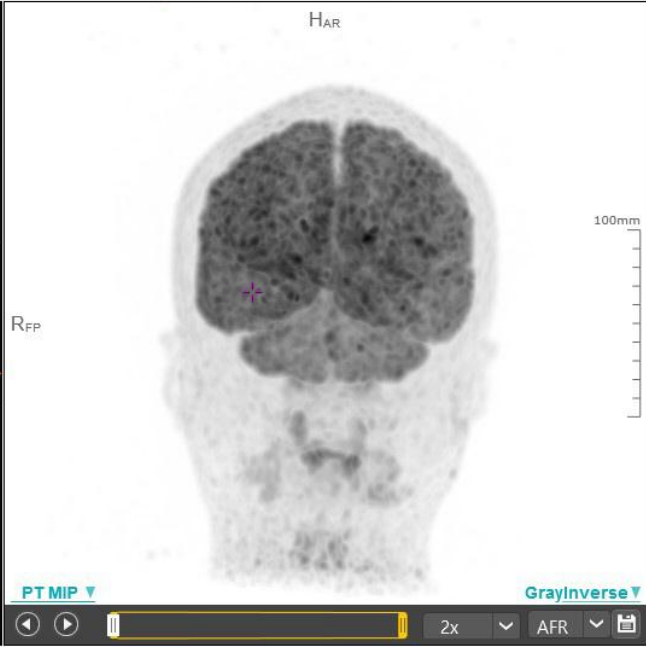

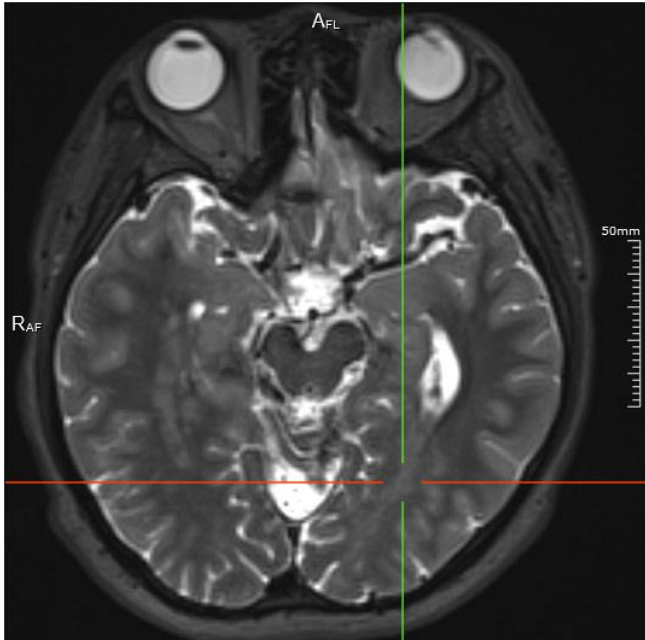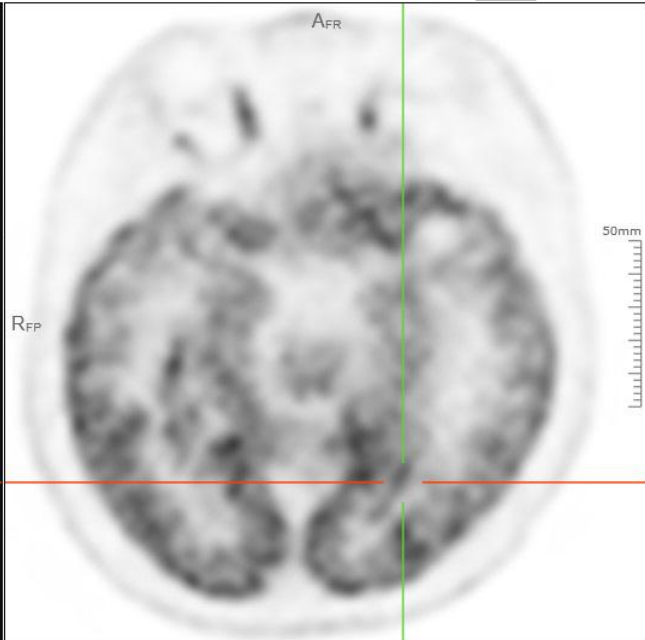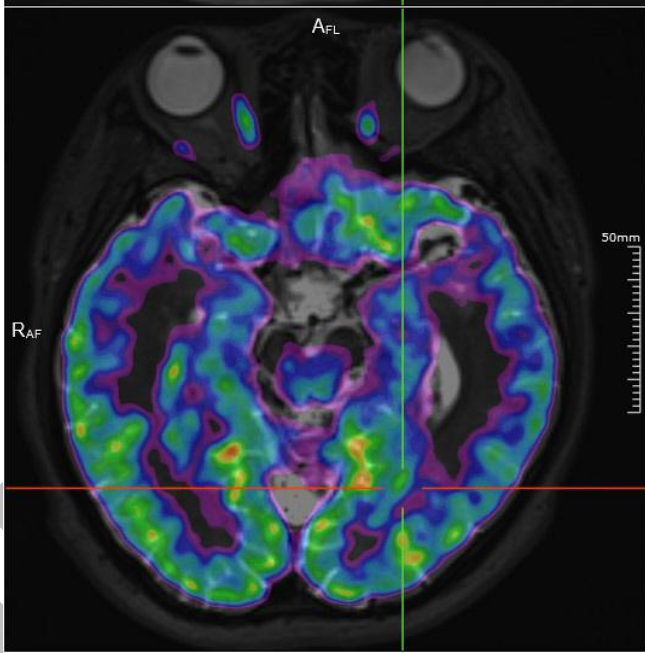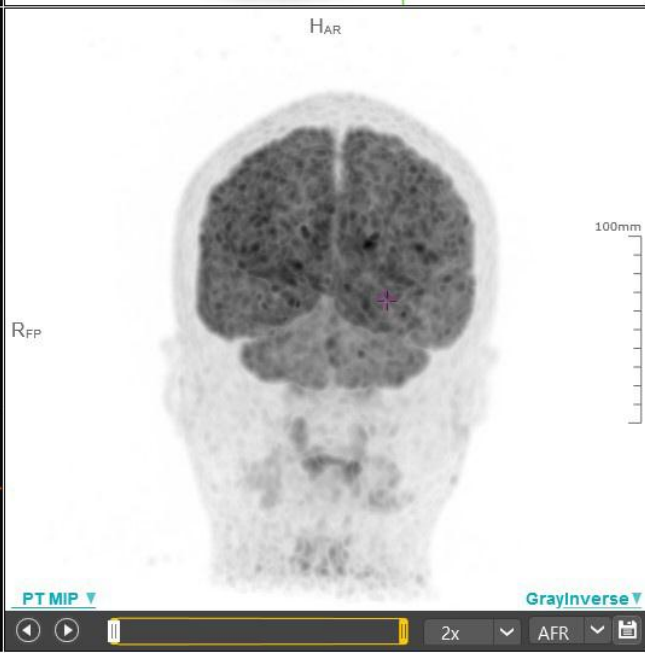

H<sub>AR</sub>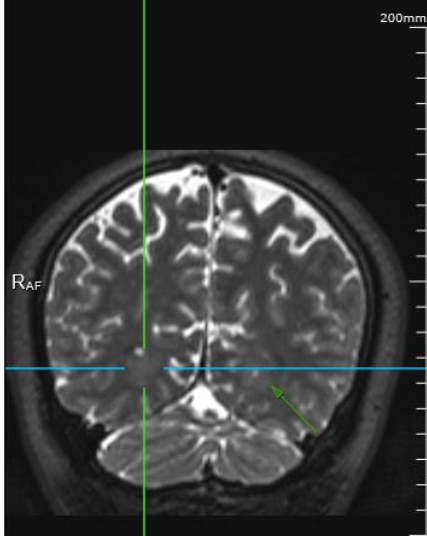H<sub>AR</sub>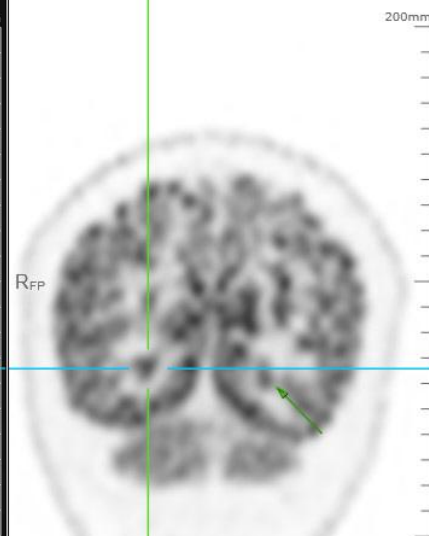H<sub>AR</sub>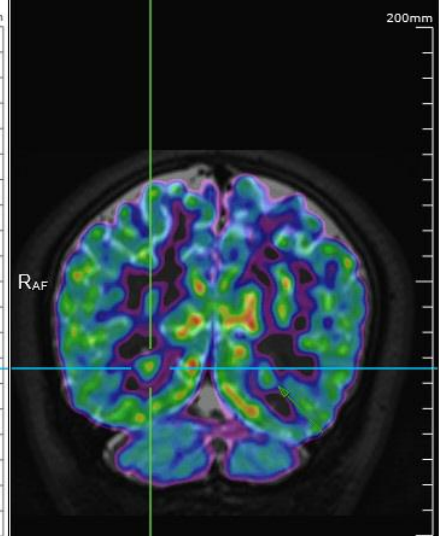

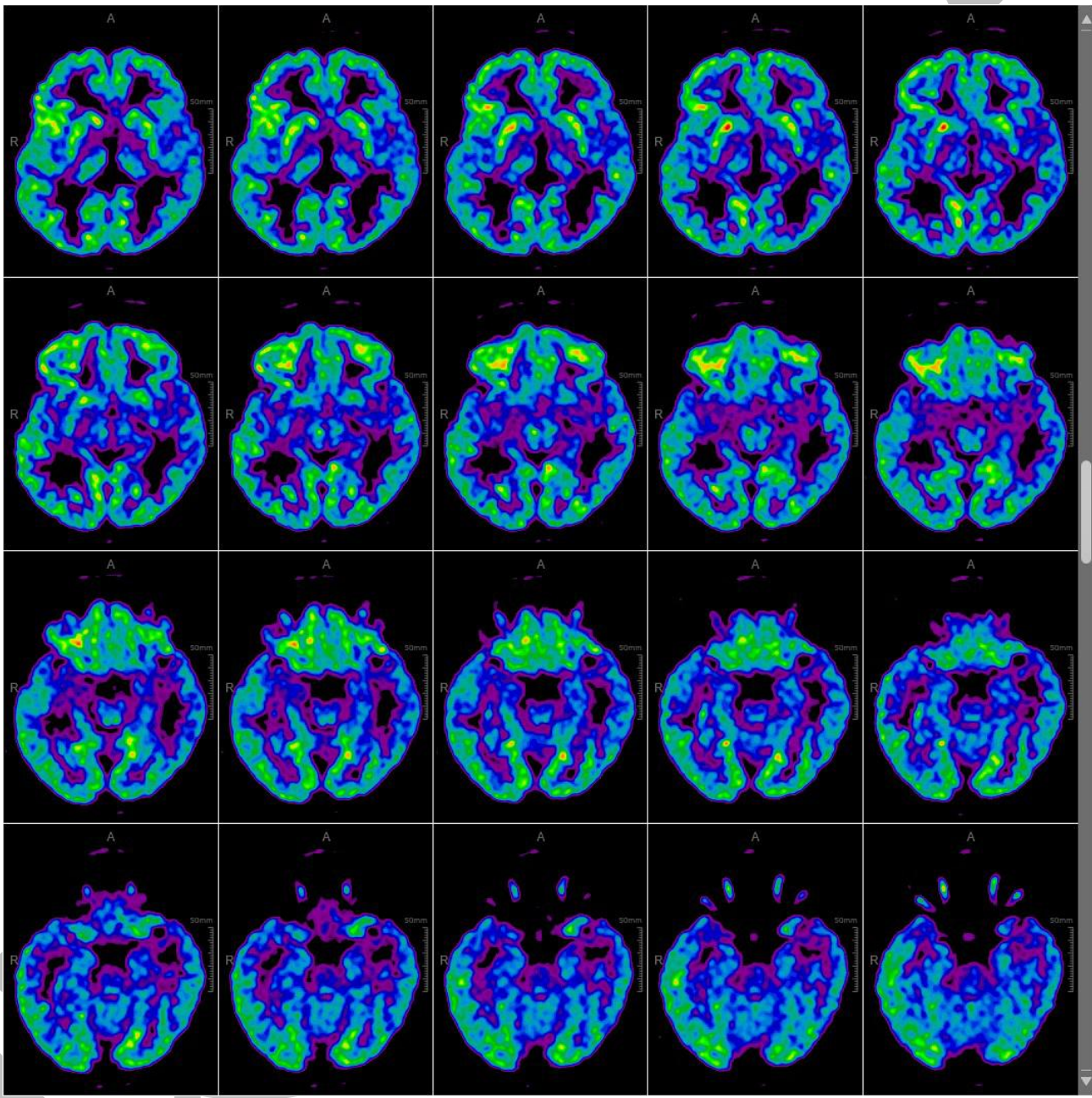

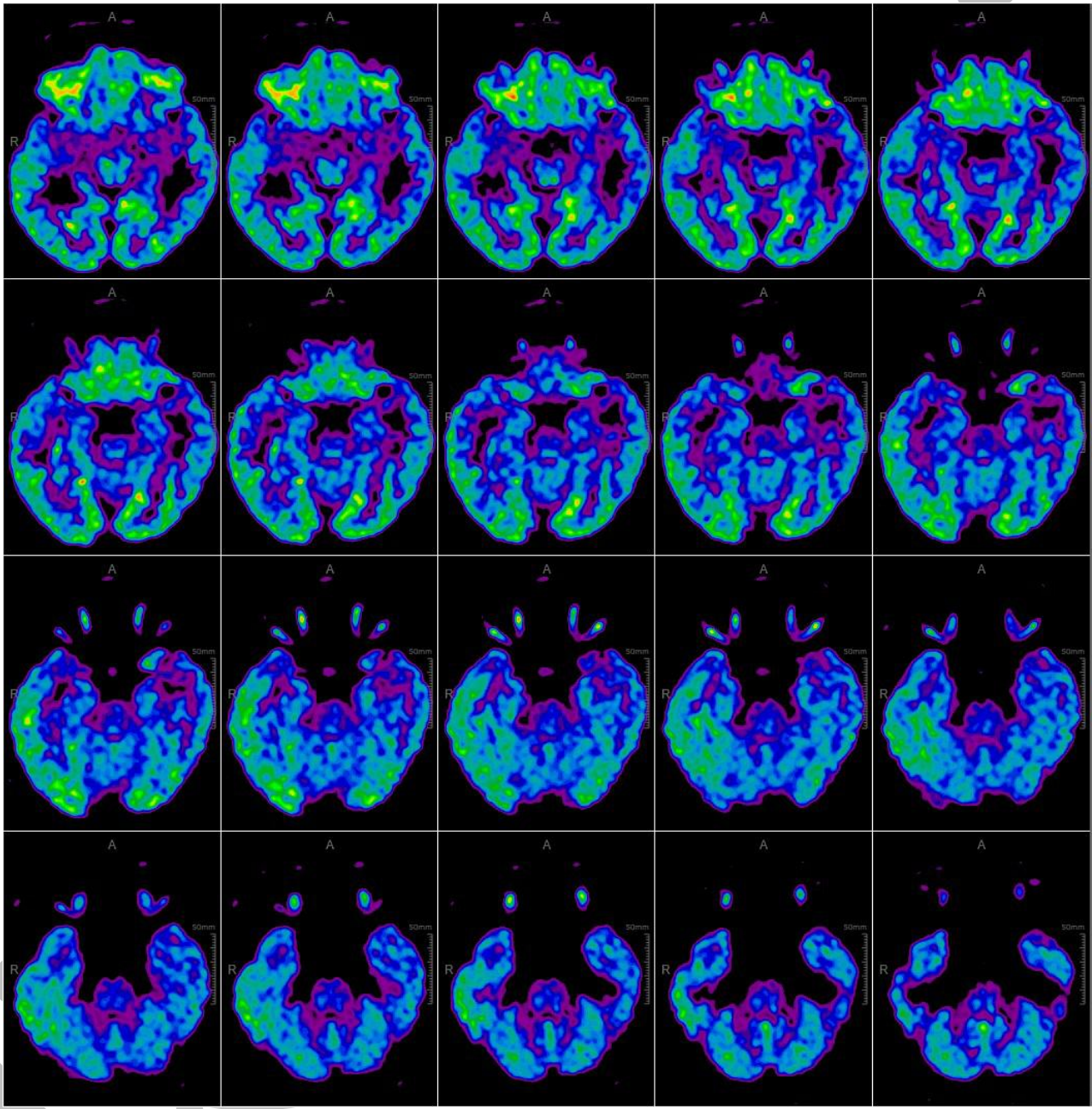

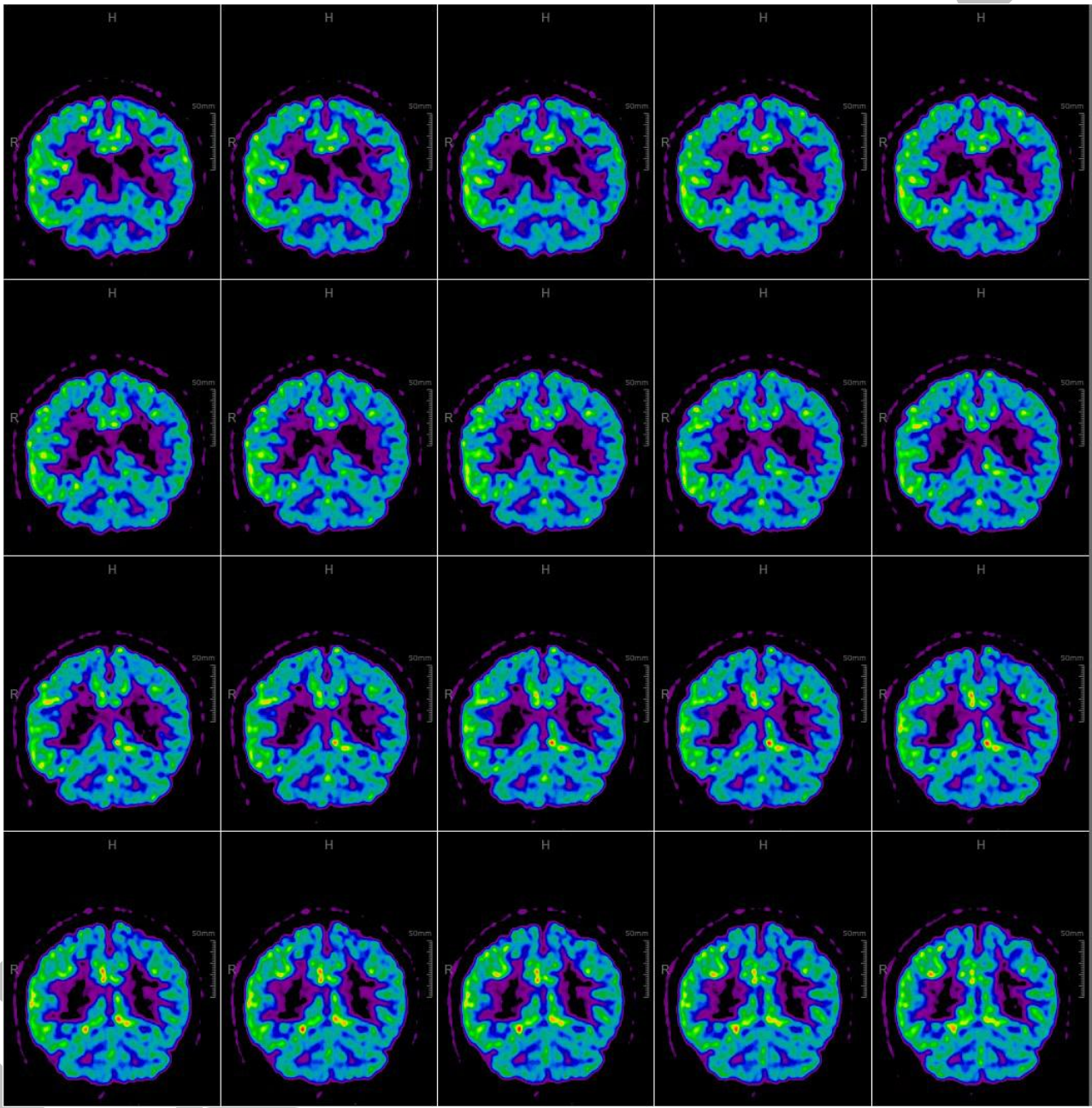

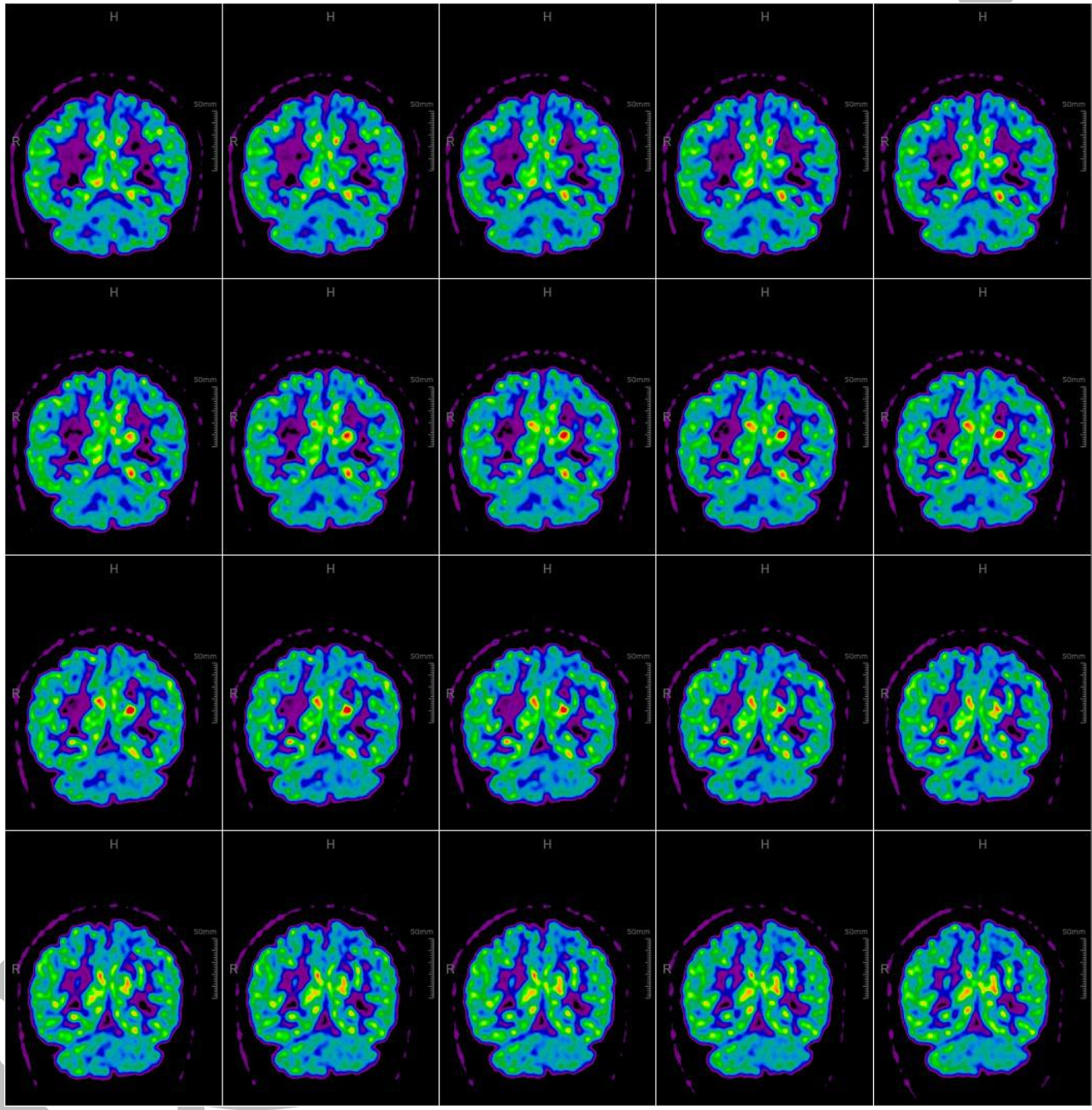

Supplement: Supplementary file 1 [file Data_Sheet_1.pdf]
