# Supplementary material for: RELN gene-related drug-resistant epilepsy with periventricular nodular heterotopia treated with radiofrequency thermocoagulation: a case report
Source: Front Neurol. 2024 Mar 27;15:1366776. doi: 10.3389/fneur.2024.1366776 (PMC11004351; doi:10.3389/fneur.2024.1366776)
Supplement: Supplementary file 2 [file Presentation_1.PPTX]

## Slide 1
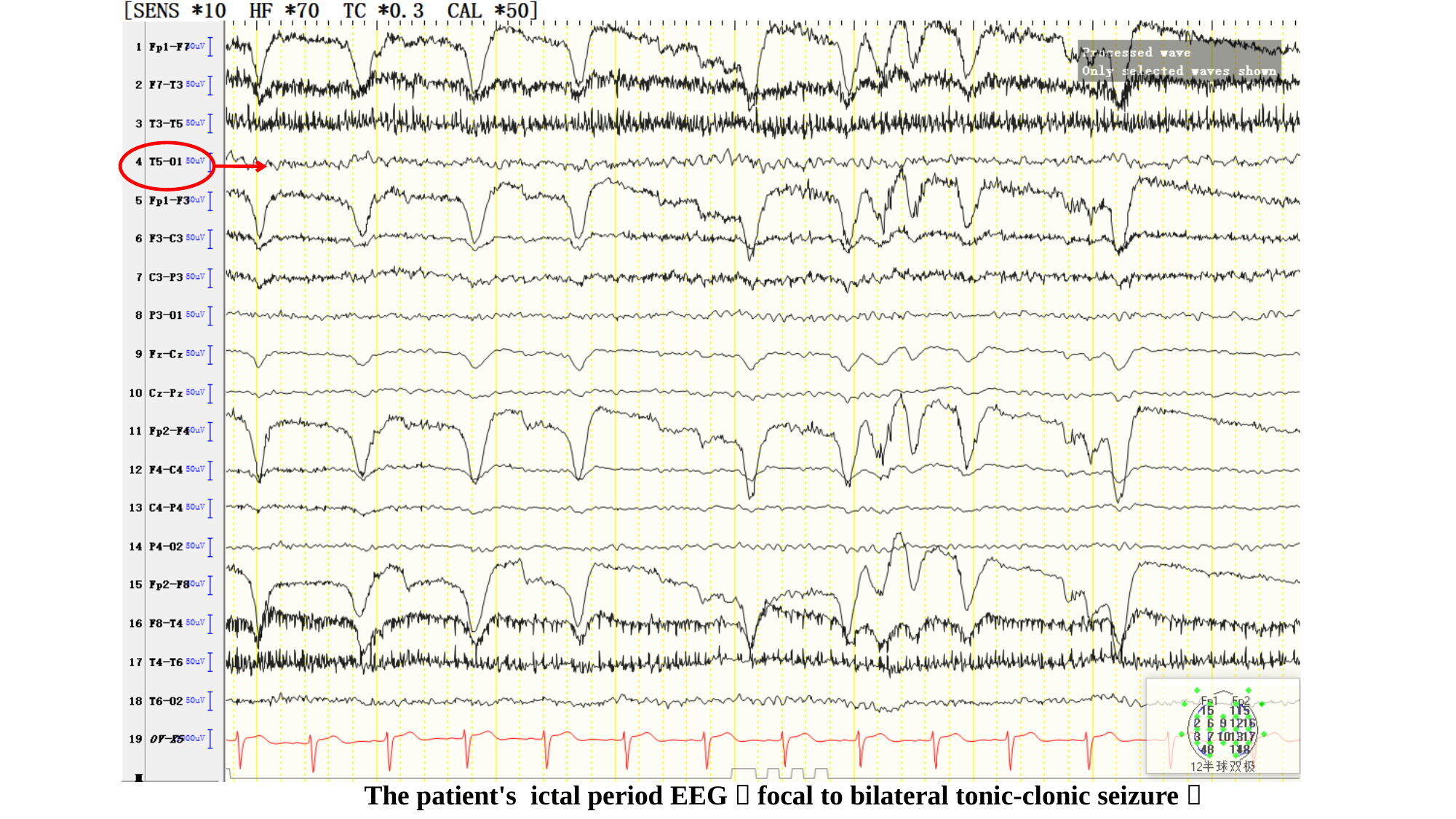

The patient's ictal period EEG（focal to bilateral tonic-clonic seizure）

## Slide 2
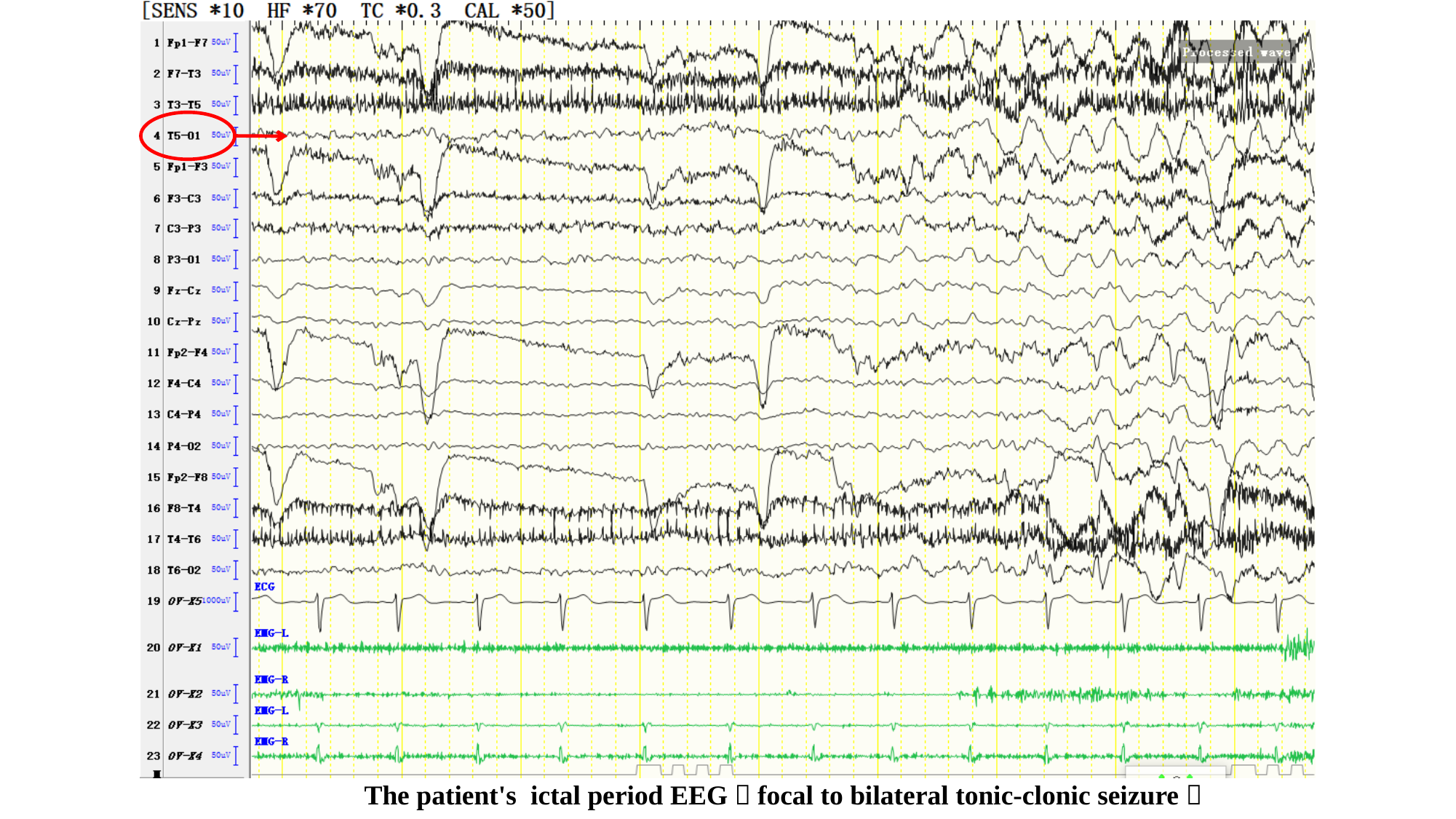

The patient's ictal period EEG（focal to bilateral tonic-clonic seizure）

## Slide 3
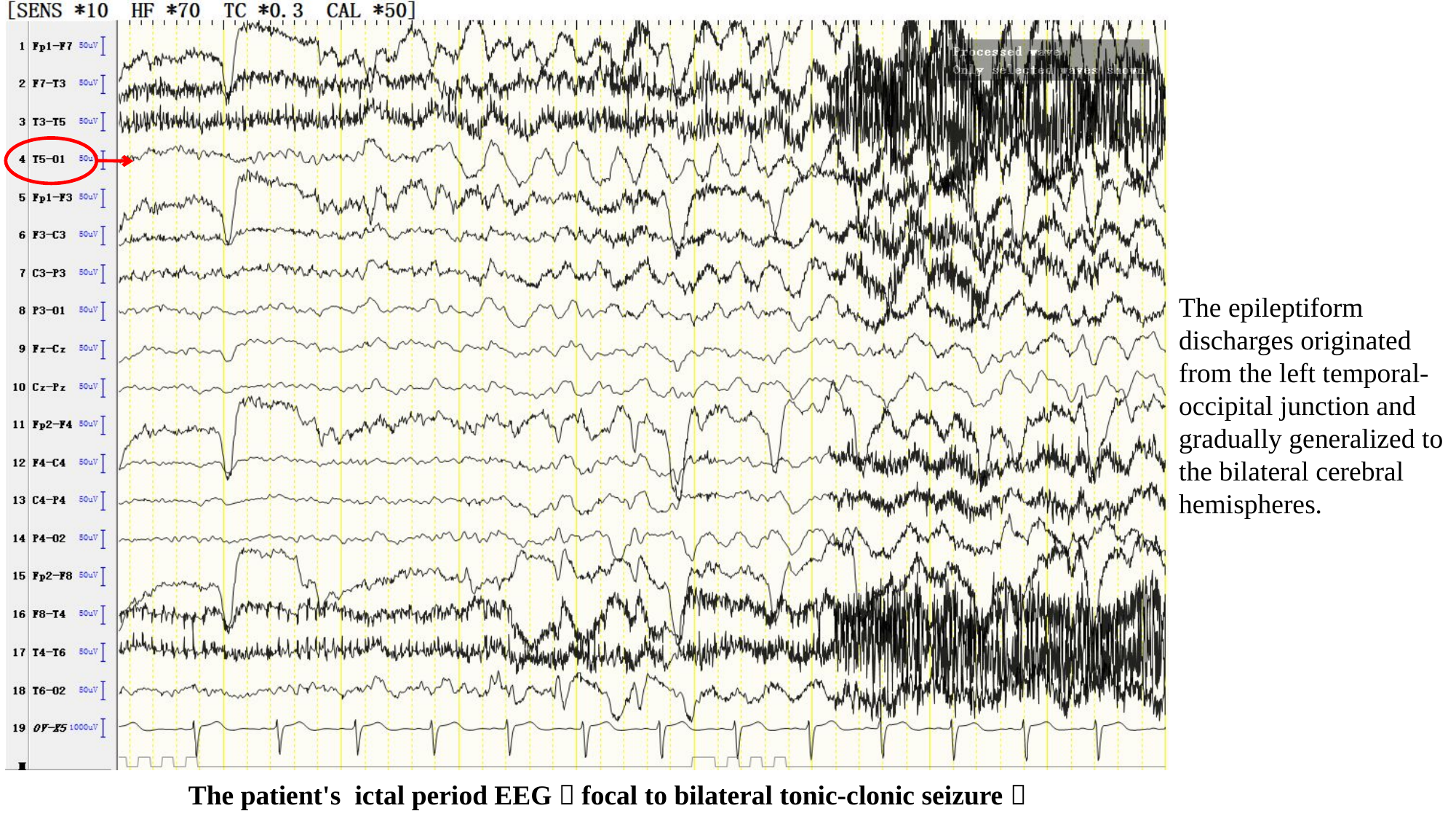

The epileptiform discharges originated from the left temporal-occipital junction and gradually generalized to the bilateral cerebral hemispheres.
The patient's ictal period EEG（focal to bilateral tonic-clonic seizure）

## Slide 4
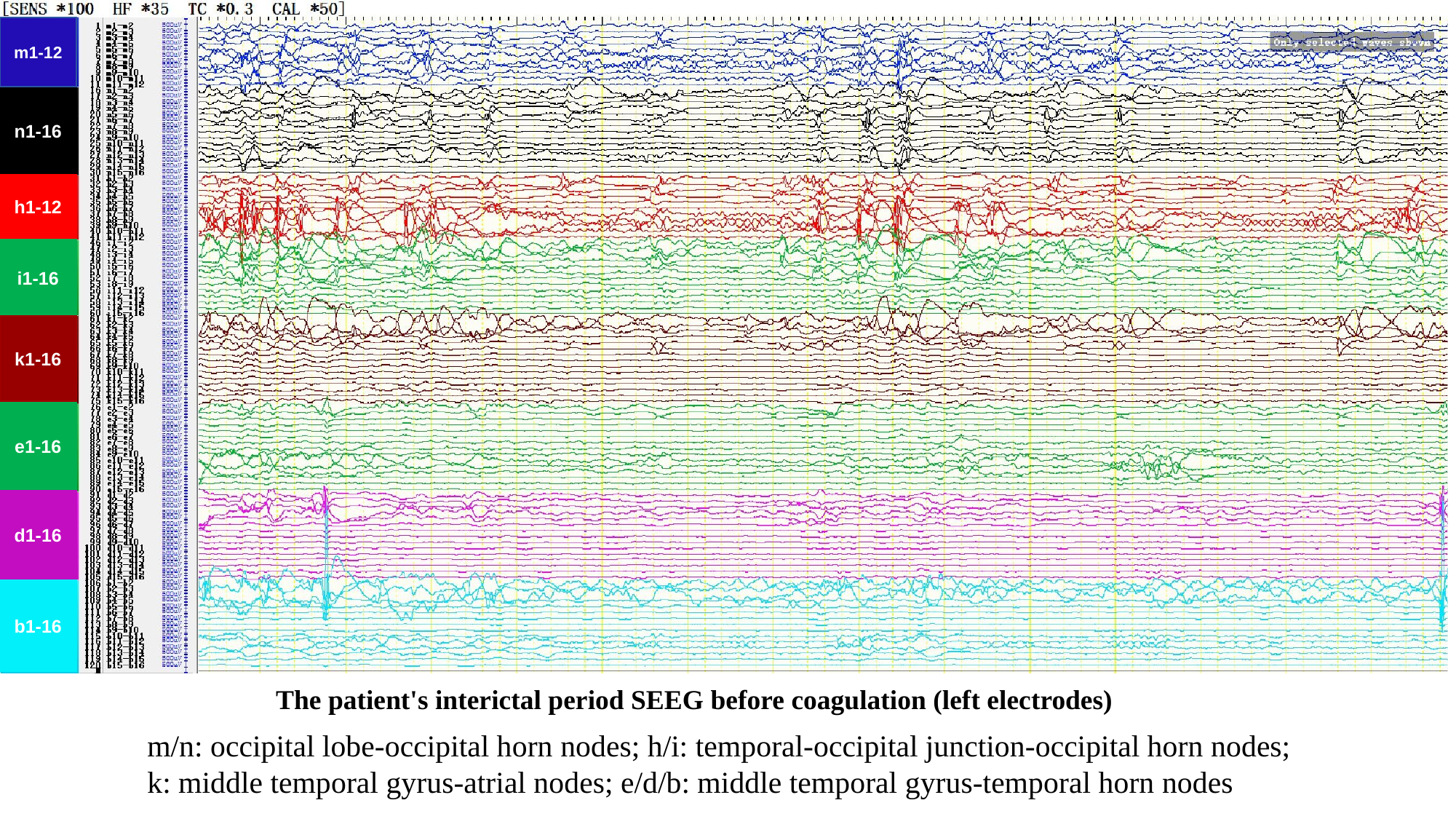

m1-12
n1-16
h1-12
i1-16
k1-16
e1-16
d1-16
b1-16
The patient's interictal period SEEG before coagulation (left electrodes)
m/n: occipital lobe-occipital horn nodes; h/i: temporal-occipital junction-occipital horn nodes; k: middle temporal gyrus-atrial nodes; e/d/b: middle temporal gyrus-temporal horn nodes

## Slide 5
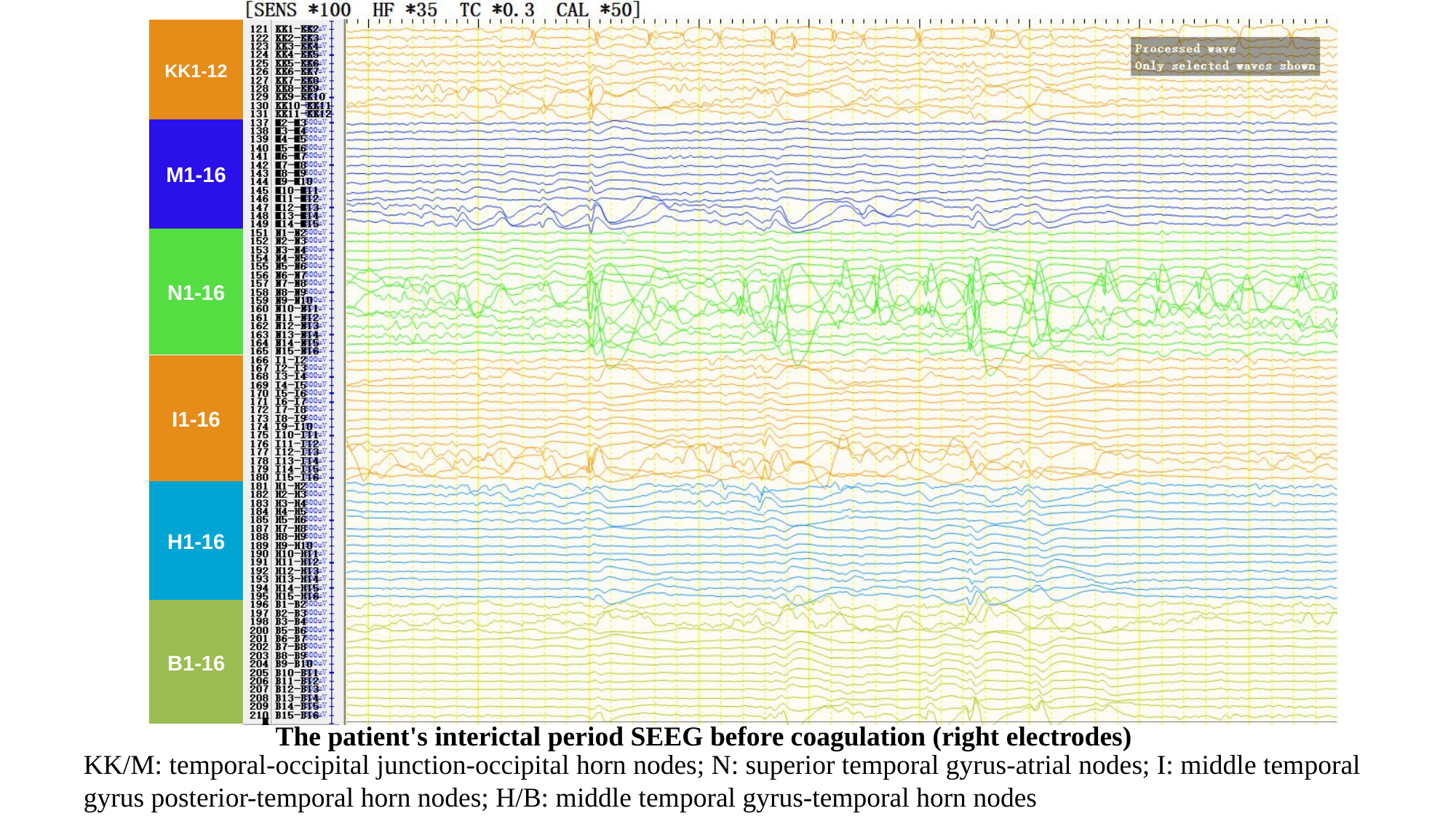

KK1-12
M1-16
N1-16
I1-16
H1-16
B1-16
The patient's interictal period SEEG before coagulation (right electrodes)
KK/M: temporal-occipital junction-occipital horn nodes; N: superior temporal gyrus-atrial nodes; I: middle temporal gyrus posterior-temporal horn nodes; H/B: middle temporal gyrus-temporal horn nodes

## Slide 6
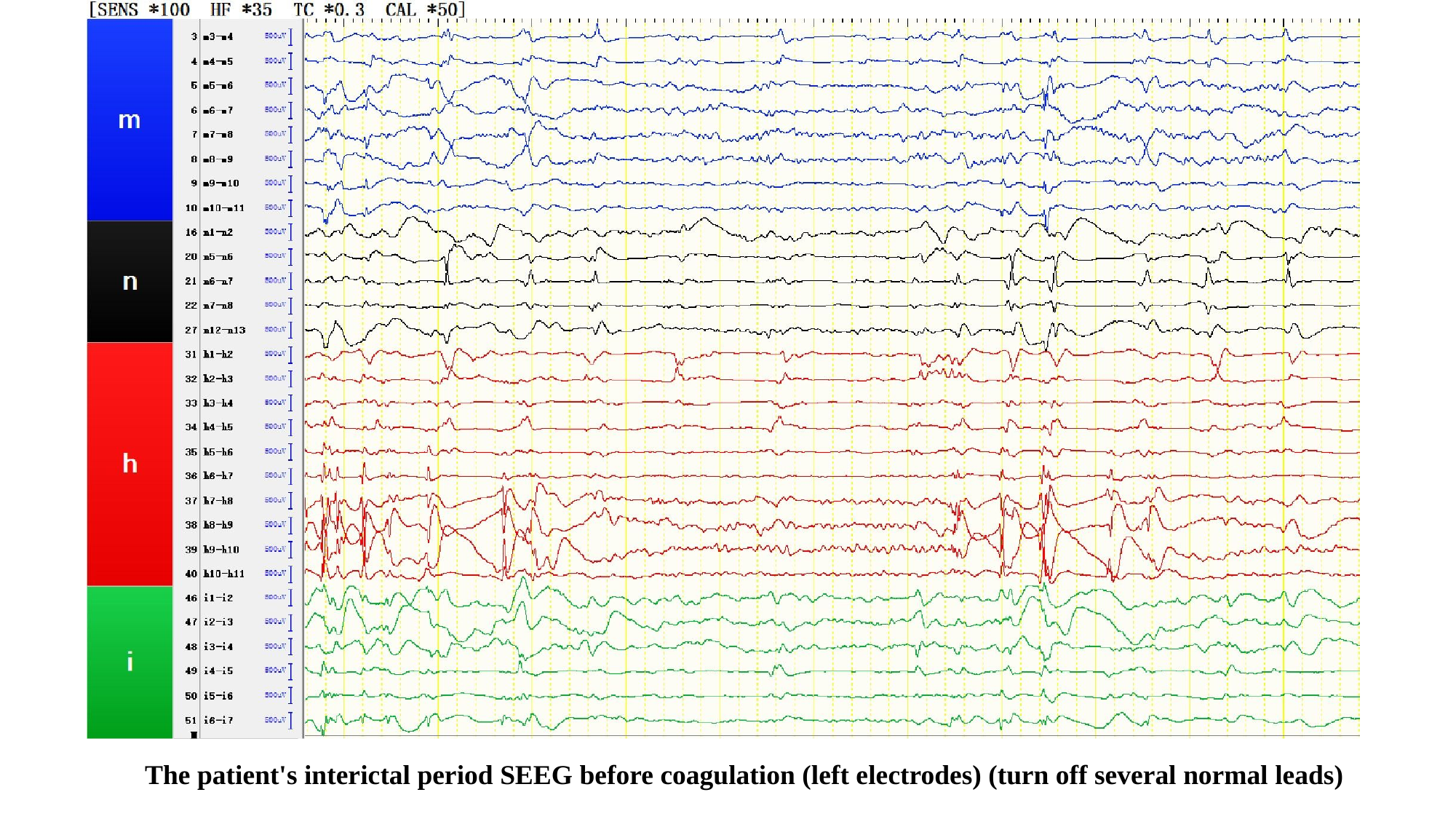

The patient's interictal period SEEG before coagulation (left electrodes) (turn off several normal leads)

## Slide 7
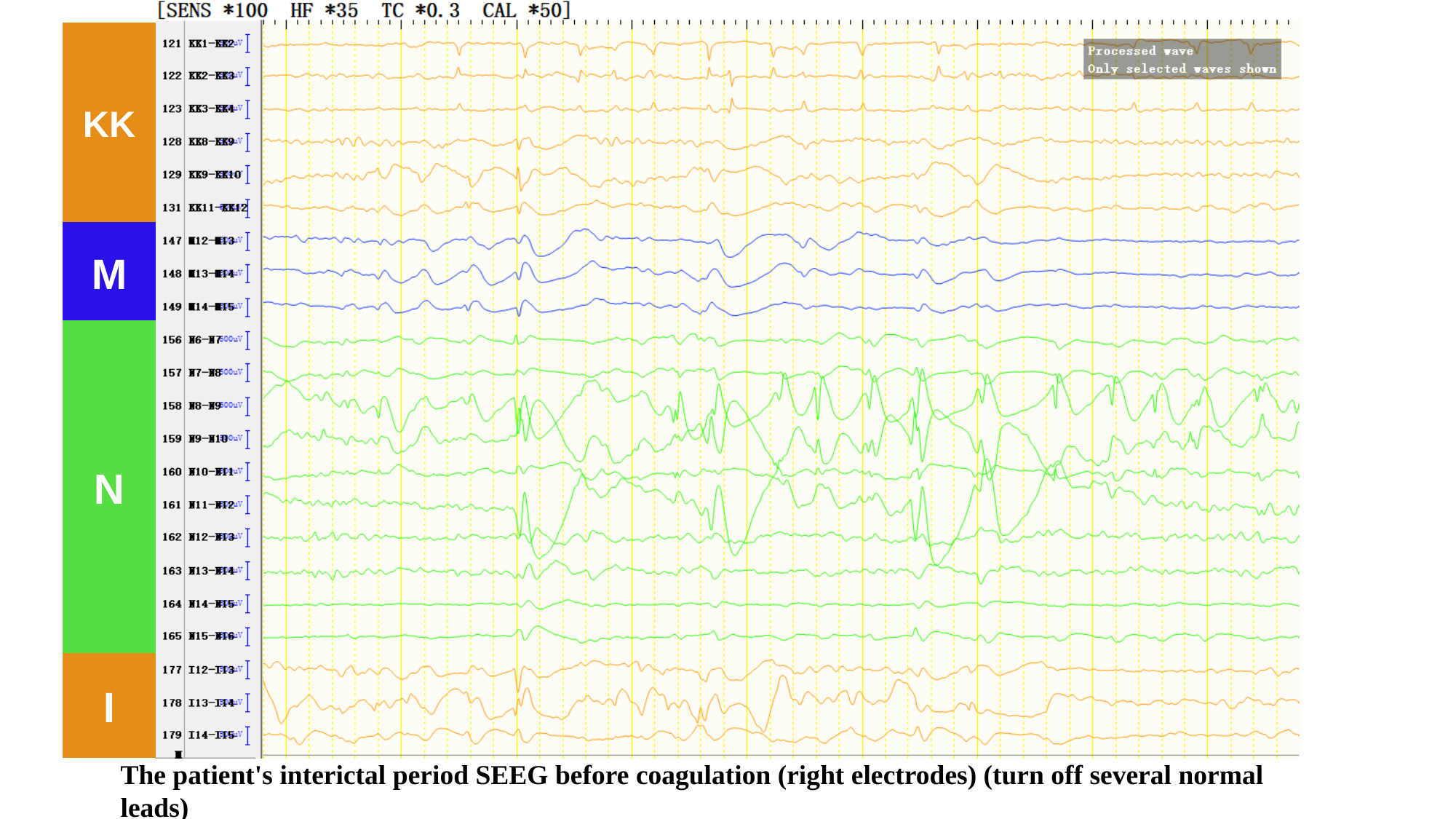

KK
M
N
I
The patient's interictal period SEEG before coagulation (right electrodes) (turn off several normal leads)

## Slide 8
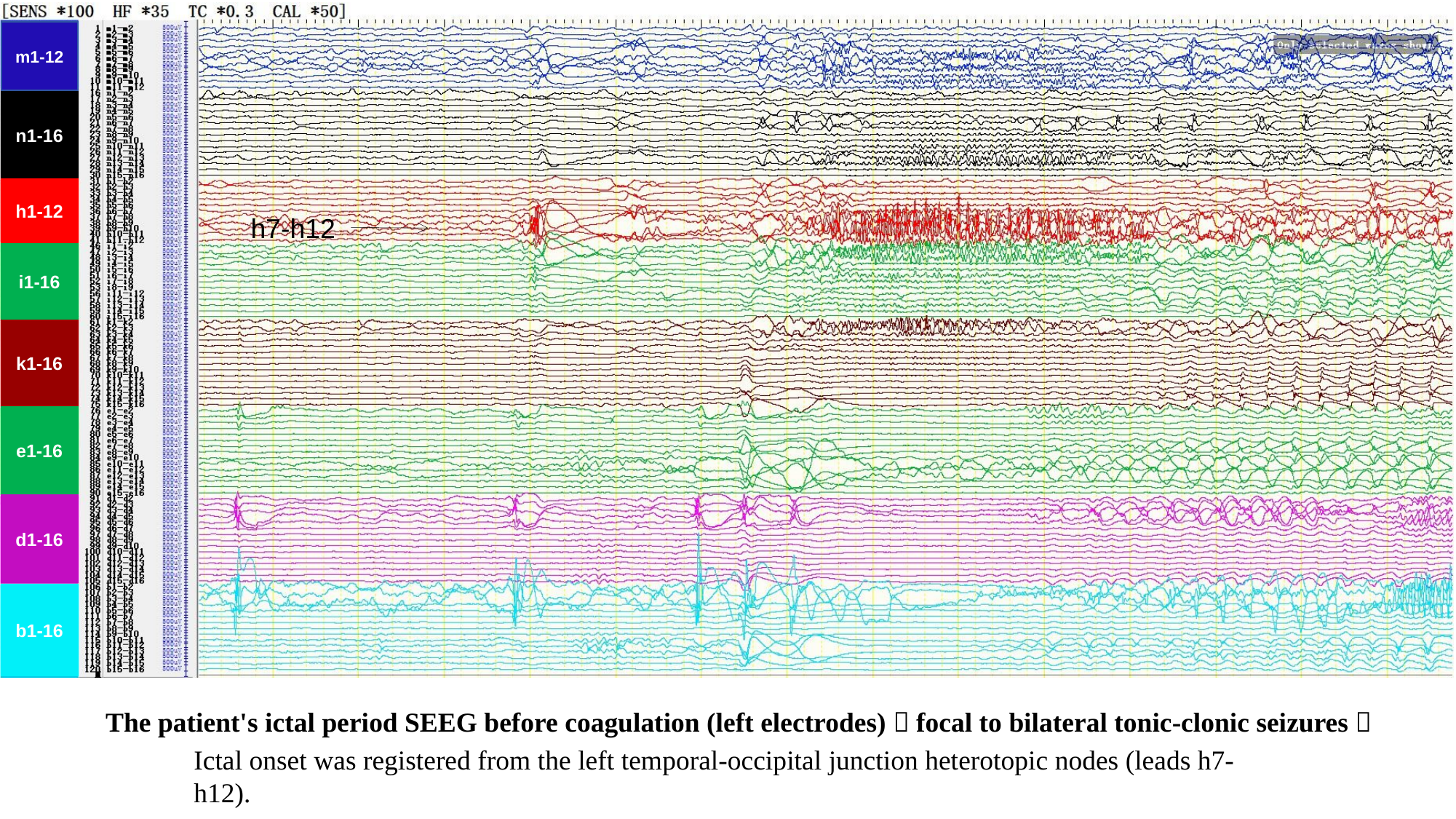

m1-12
n1-16
h1-12
i1-16
k1-16
e1-16
d1-16
b1-16
h7-h12
The patient's ictal period SEEG before coagulation (left electrodes)（focal to bilateral tonic-clonic seizures）
Ictal onset was registered from the left temporal-occipital junction heterotopic nodes (leads h7-h12).

## Slide 9
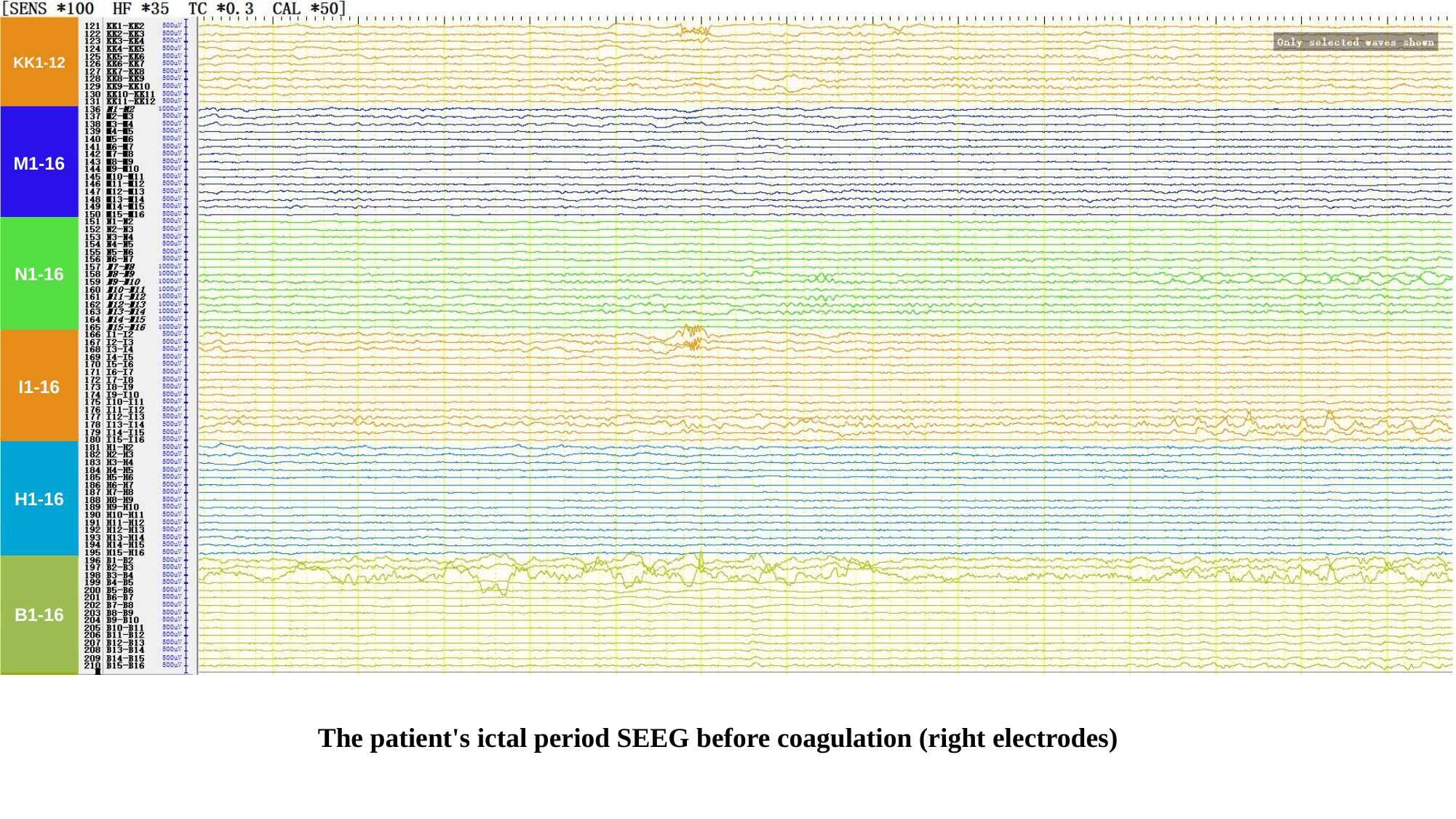

KK1-12
M1-16
N1-16
I1-16
H1-16
B1-16
The patient's ictal period SEEG before coagulation (right electrodes)

## Slide 10
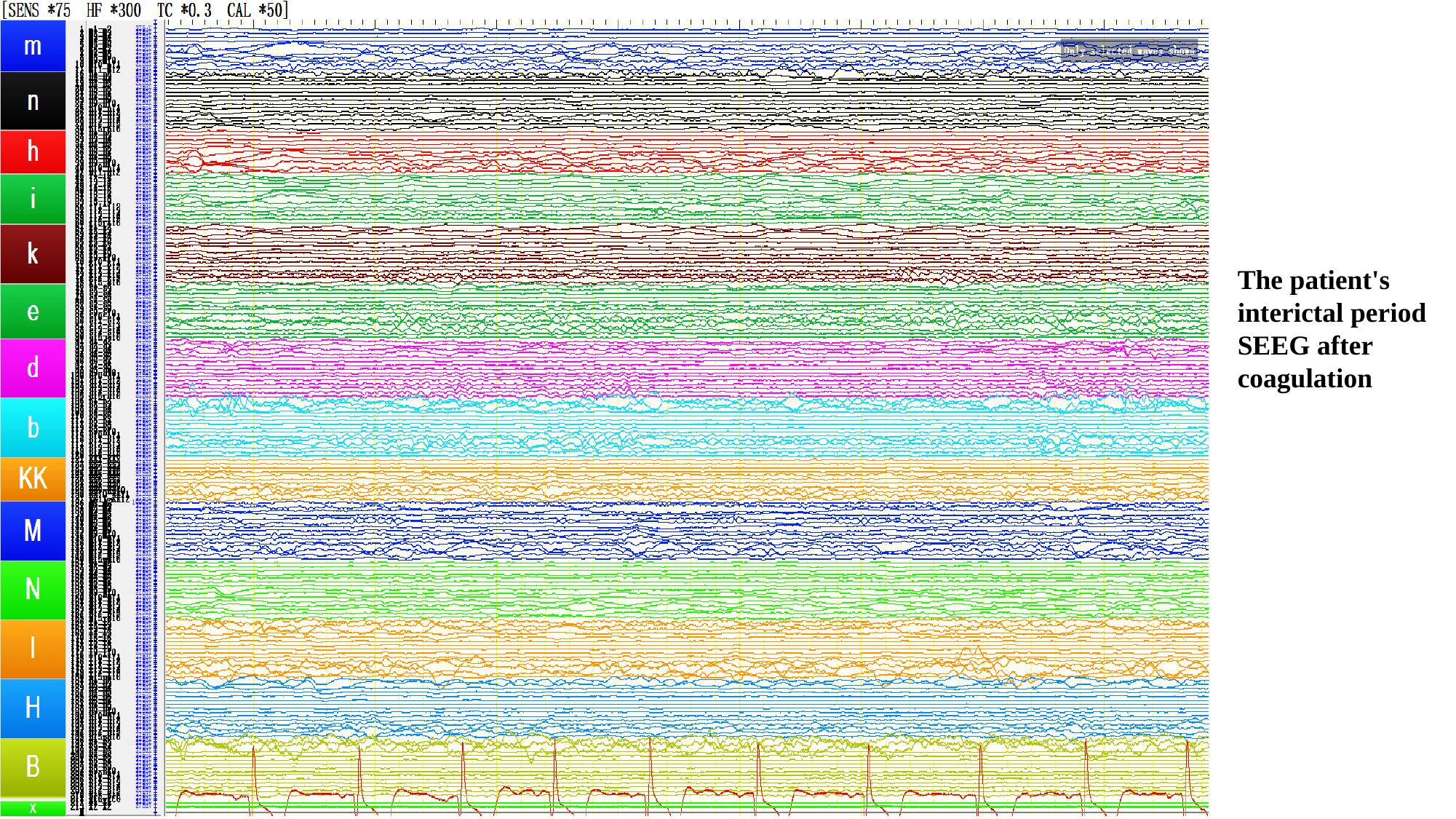

The patient's interictal period SEEG after coagulation

## Slide 11
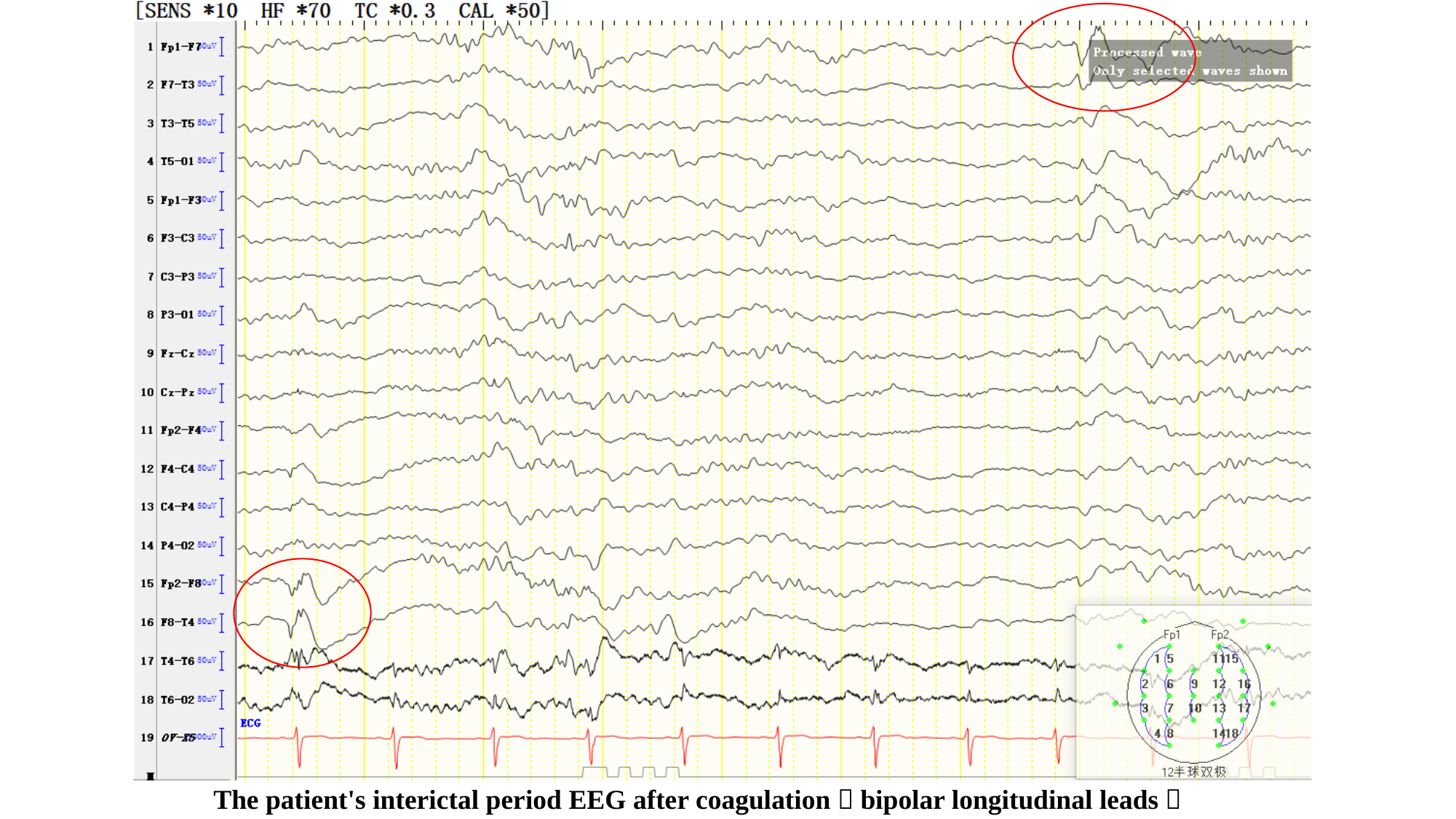

The patient's interictal period EEG after coagulation（bipolar longitudinal leads）
