# Supplementary material for: RELN gene-related drug-resistant epilepsy with periventricular nodular heterotopia treated with radiofrequency thermocoagulation: a case report
Source: Front Neurol. 2024 Mar 27;15:1366776. doi: 10.3389/fneur.2024.1366776 (PMC11004351; doi:10.3389/fneur.2024.1366776)
Supplement: Supplementary file 3 [file Presentation_2.PPTX]

## Slide 1
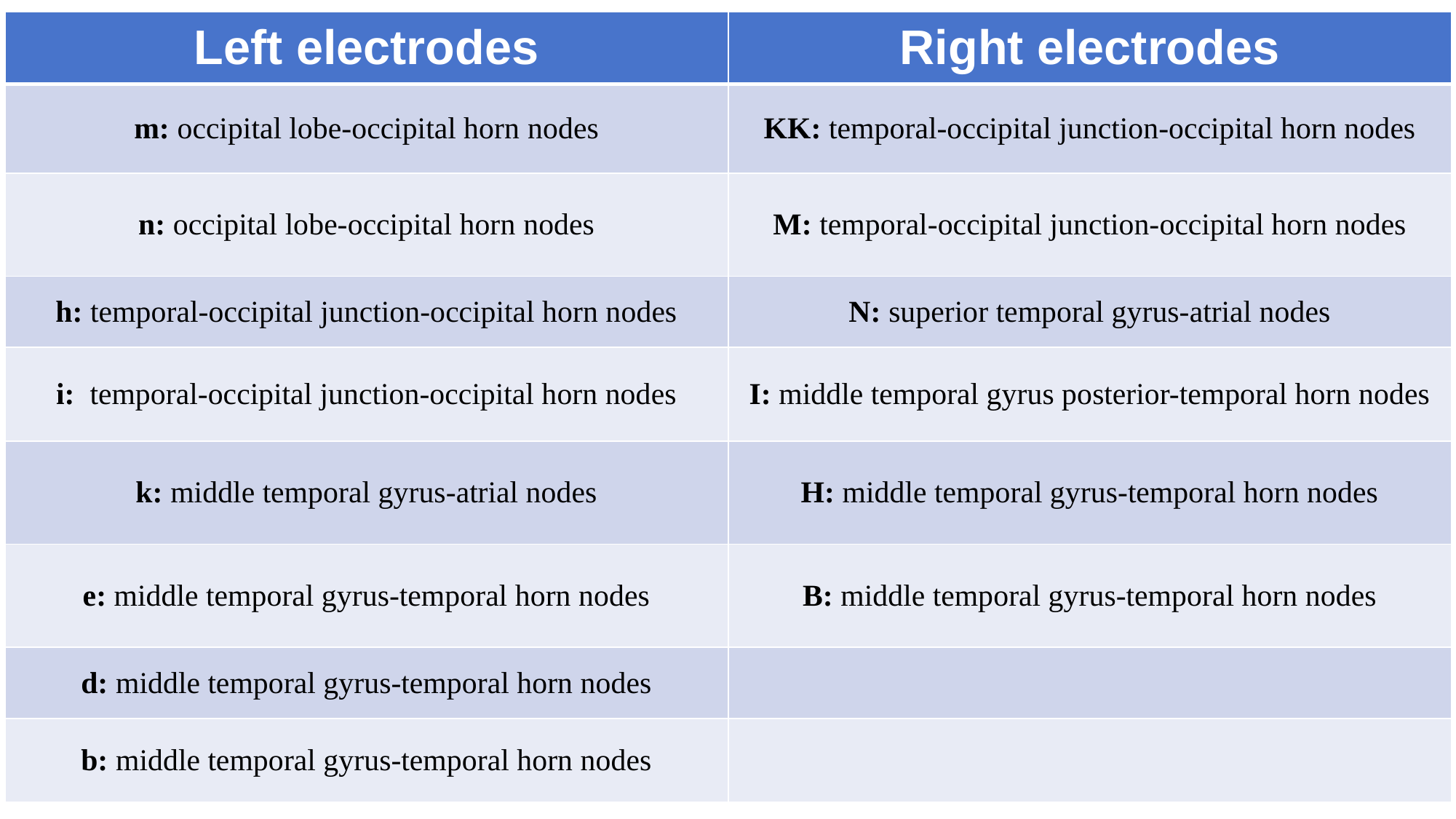

| Left electrodes | Right electrodes |
| --- | --- |
| m: occipital lobe-occipital horn nodes | KK: temporal-occipital junction-occipital horn nodes |
| n: occipital lobe-occipital horn nodes | M: temporal-occipital junction-occipital horn nodes |
| h: temporal-occipital junction-occipital horn nodes | N: superior temporal gyrus-atrial nodes |
| i: temporal-occipital junction-occipital horn nodes | I: middle temporal gyrus posterior-temporal horn nodes |
| k: middle temporal gyrus-atrial nodes | H: middle temporal gyrus-temporal horn nodes |
| e: middle temporal gyrus-temporal horn nodes | B: middle temporal gyrus-temporal horn nodes |
| d: middle temporal gyrus-temporal horn nodes | |
| b: middle temporal gyrus-temporal horn nodes | |

## Slide 2
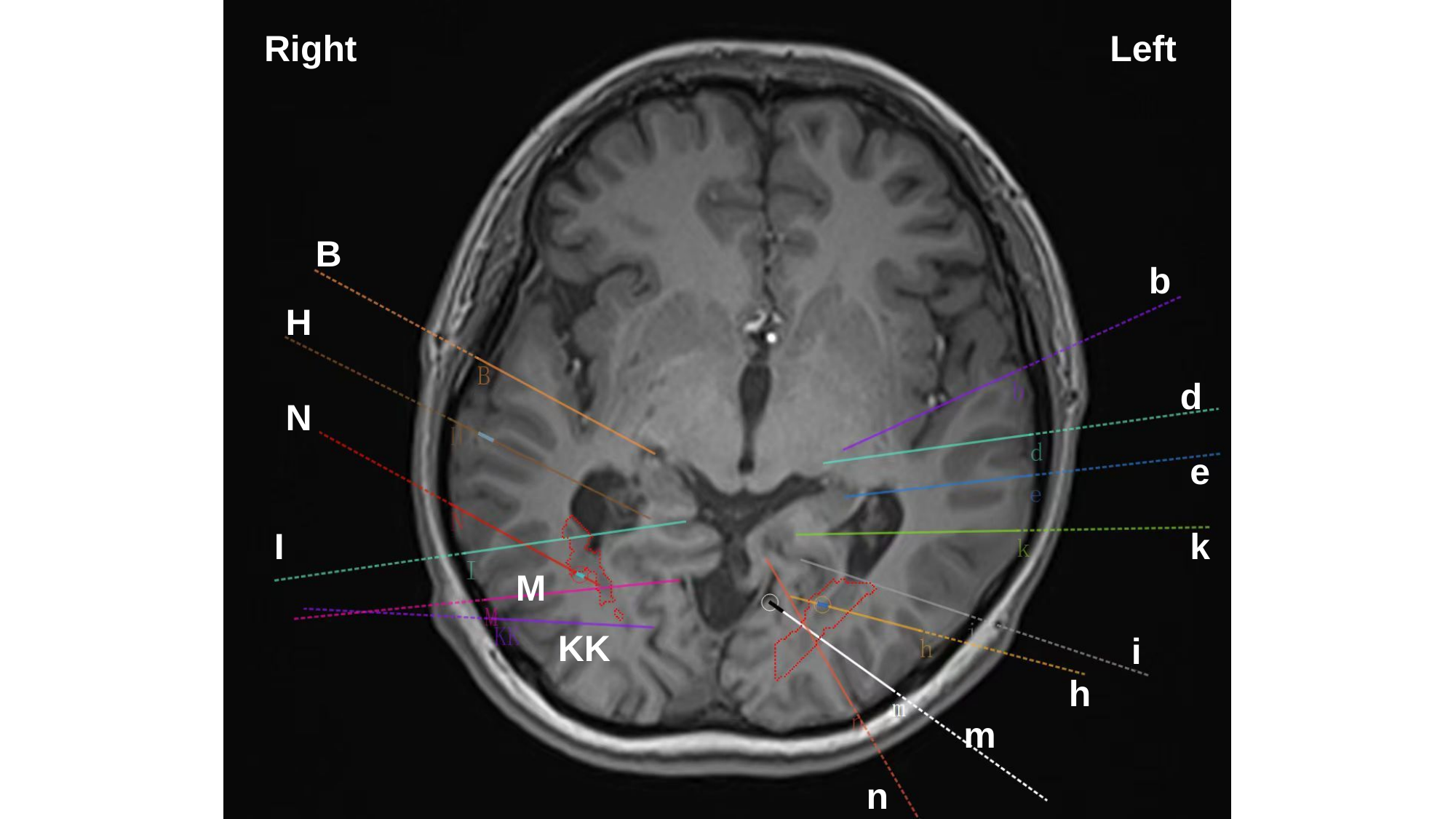

B
b
H
d
N
e
I
k
M
KK
i
h
m
n
Right
Left

## Slide 3
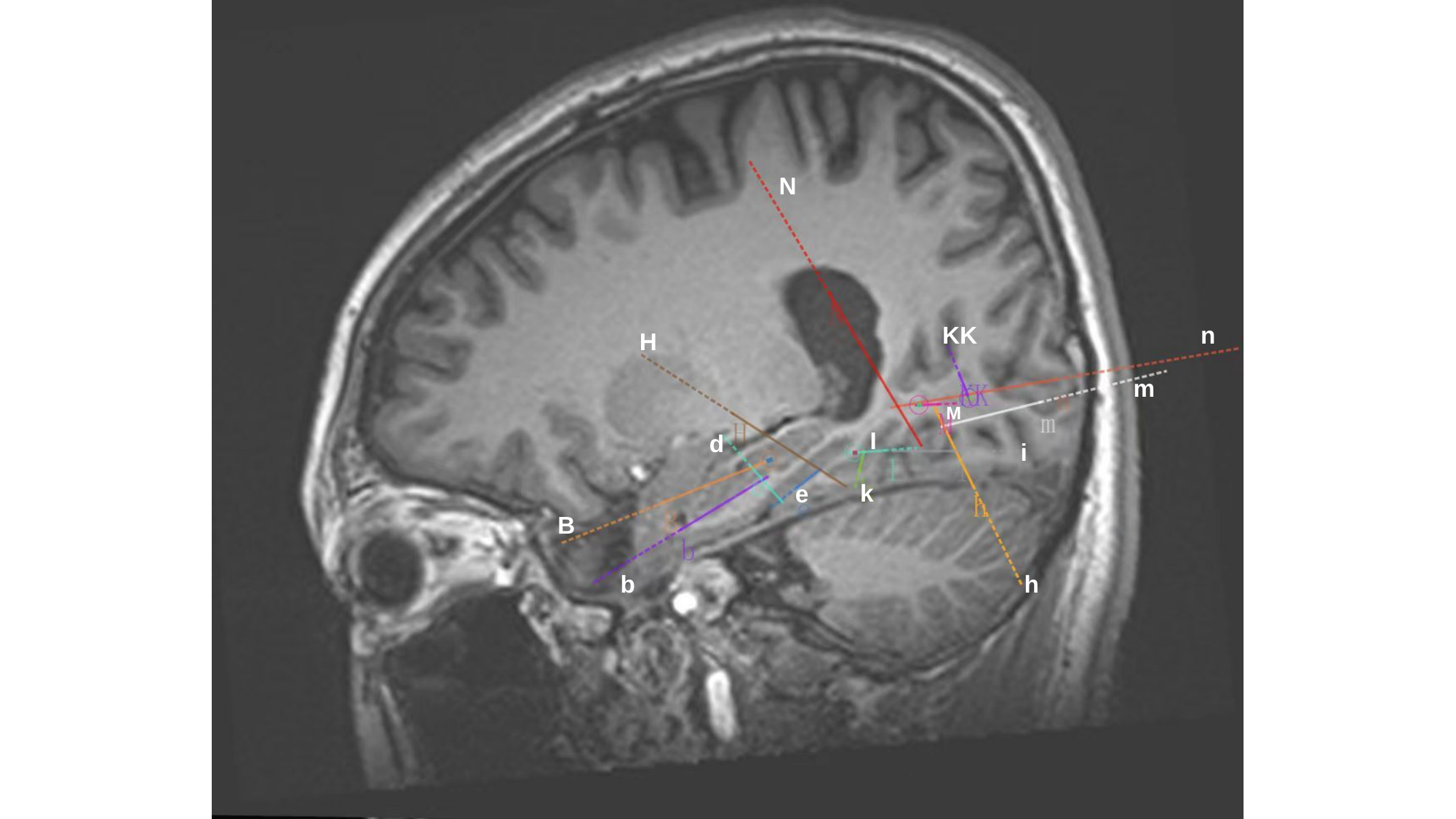

N
KK
n
H
m
M
I
d
i
k
e
B
b
h

## Slide 4
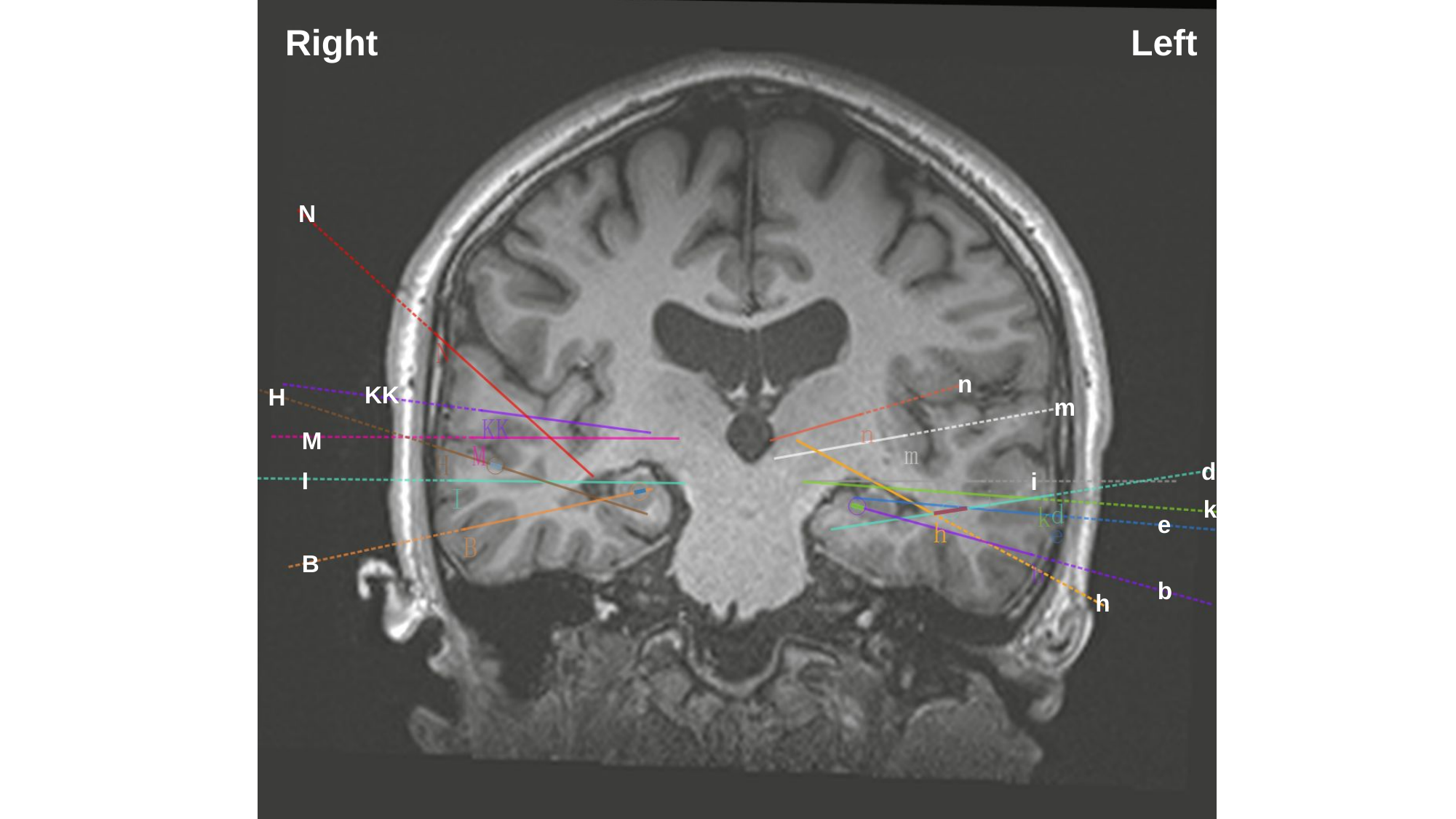

Right
Left
N
n
KK
H
m
M
d
I
i
k
e
B
b
h

## Slide 5
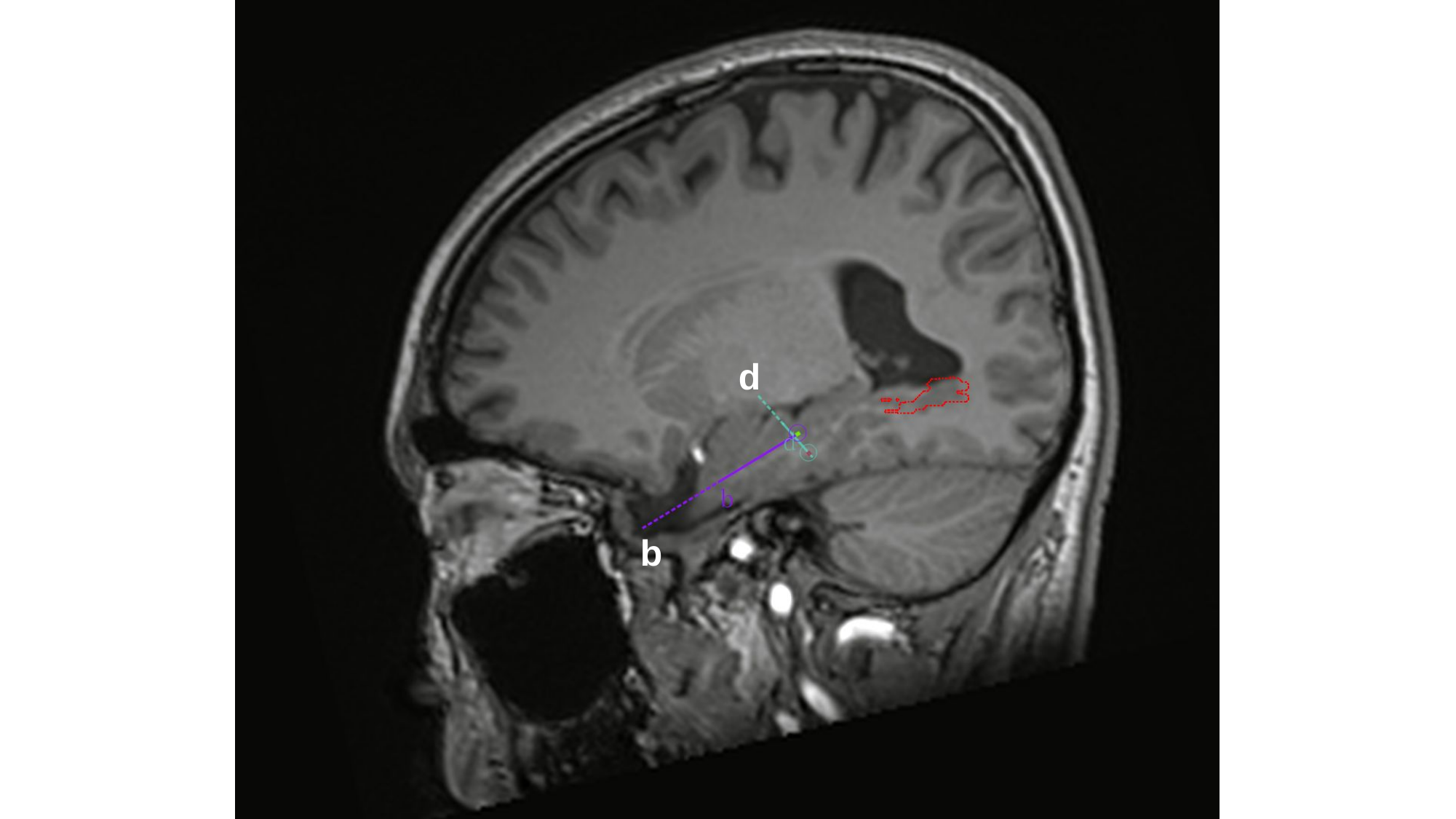

d
b

## Slide 6
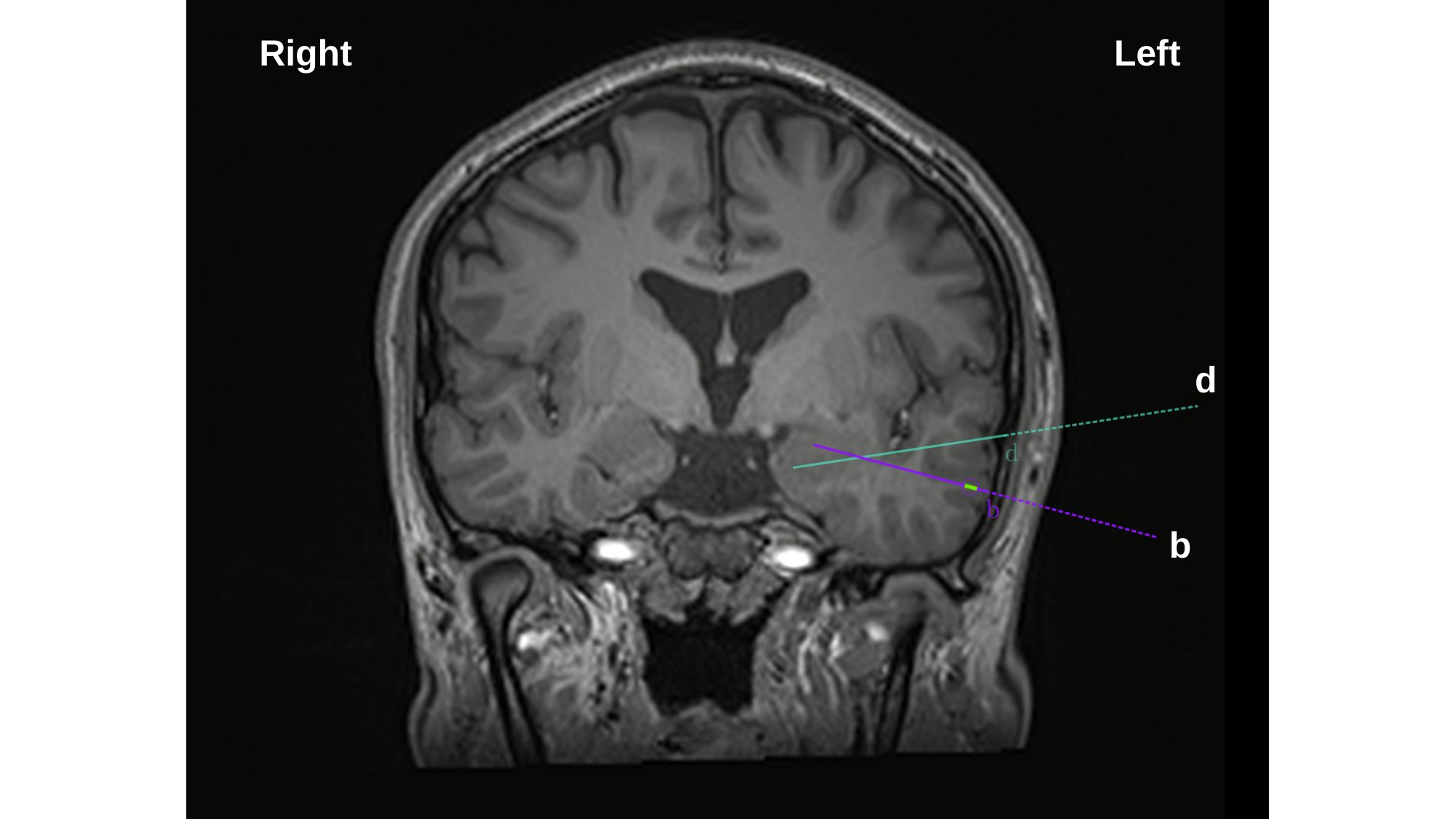

Right
Left
d
b

## Slide 7
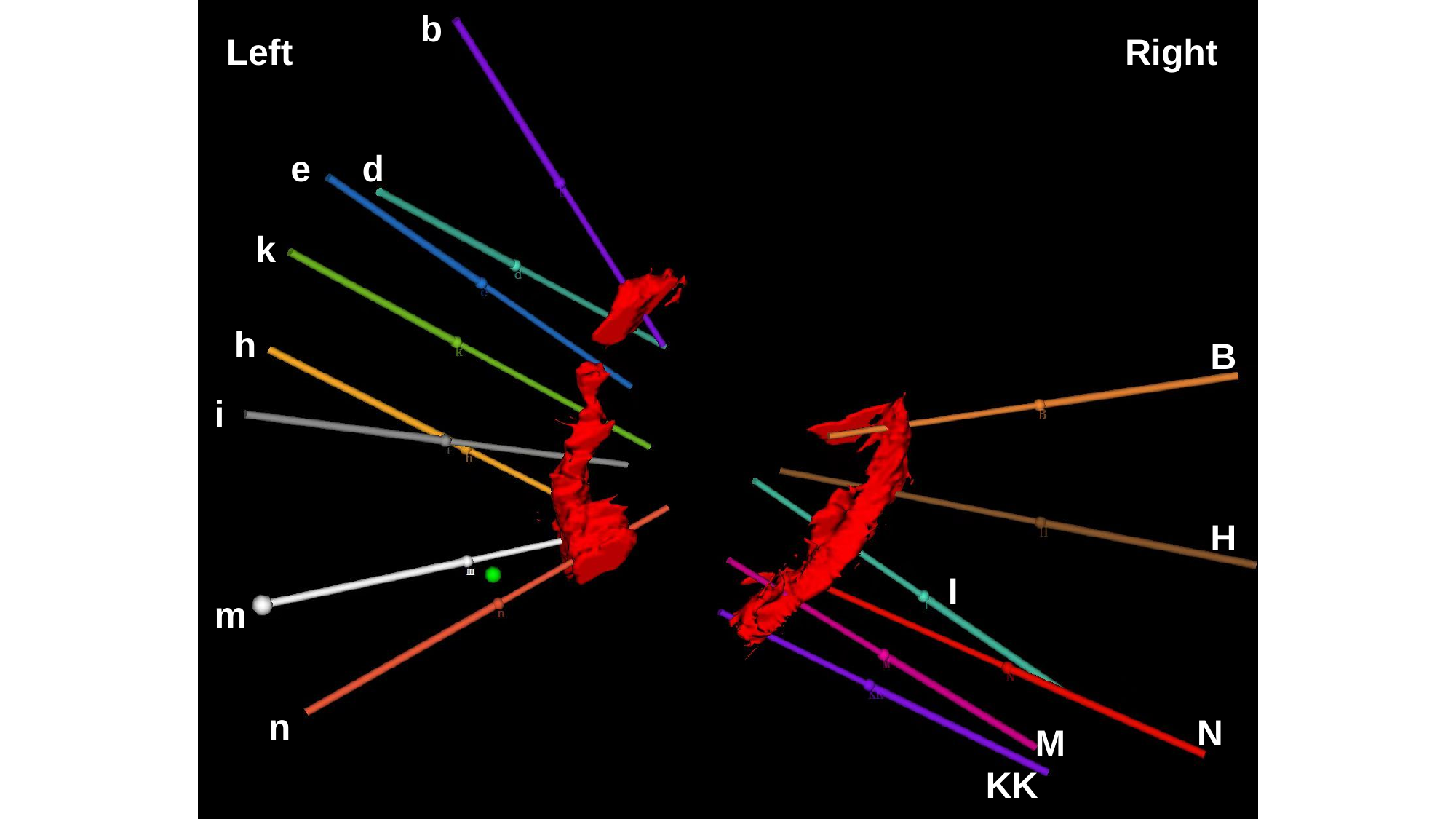

b
e
d
k
h
B
i
H
I
m
n
N
M
KK
Left
Right

## Slide 8
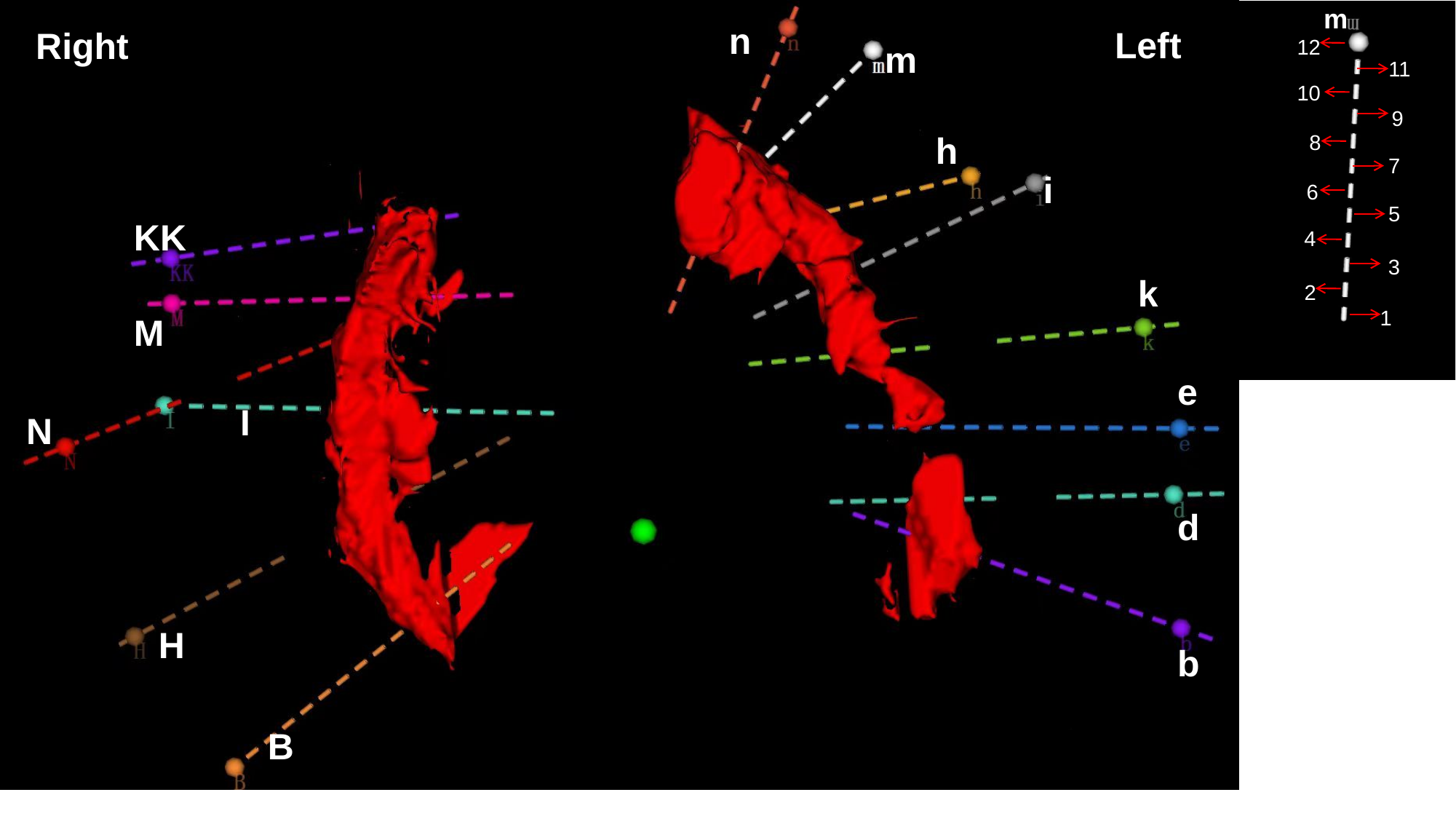

n
m
h
i
KK
k
M
e
I
N
d
H
b
B
Left
Right
m
12
11
10
9
8
7
6
5
4
3
2
1

## Slide 9
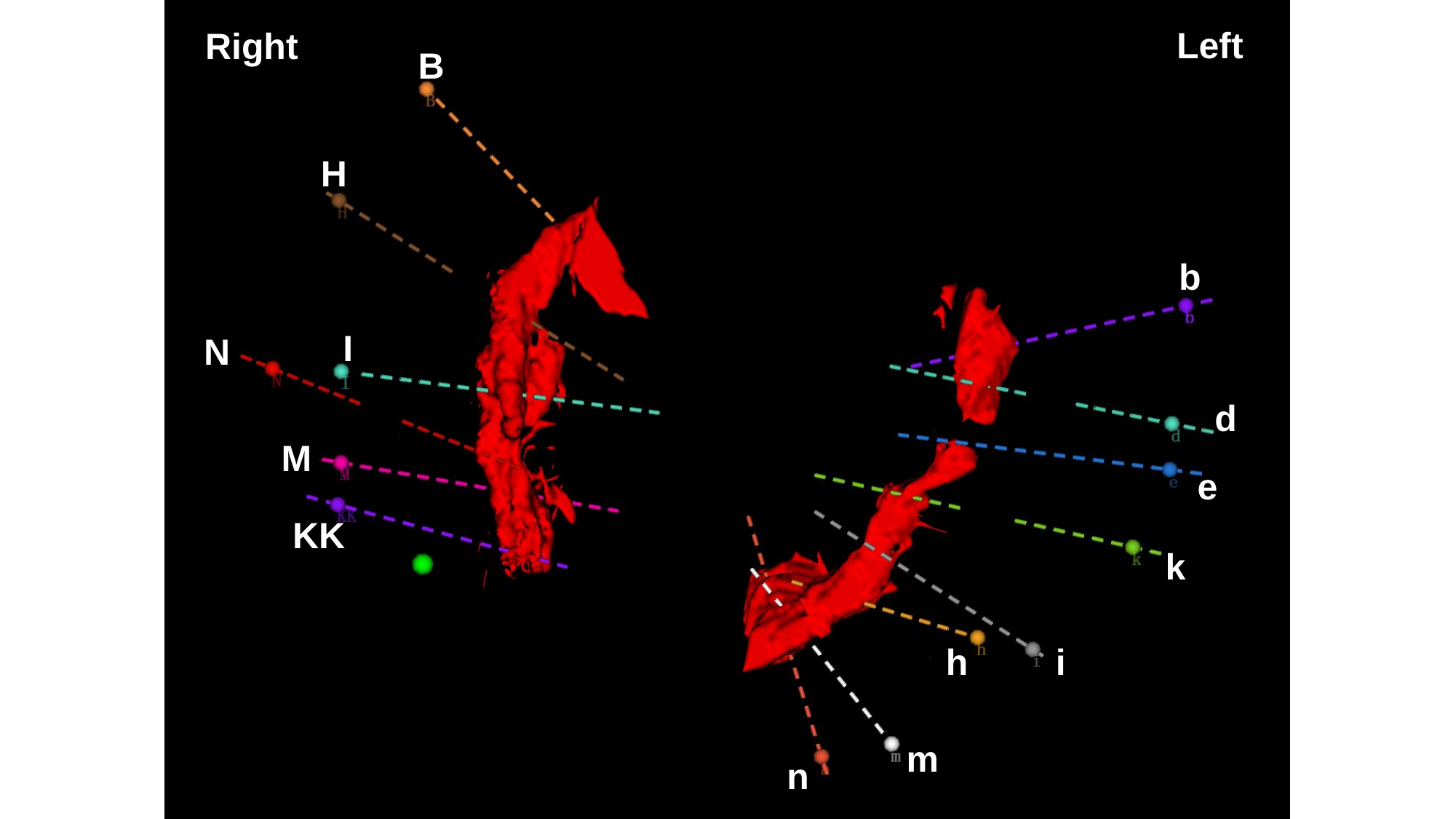

B
H
b
I
N
d
M
e
KK
k
h
i
m
n
Left
Right

## Slide 10
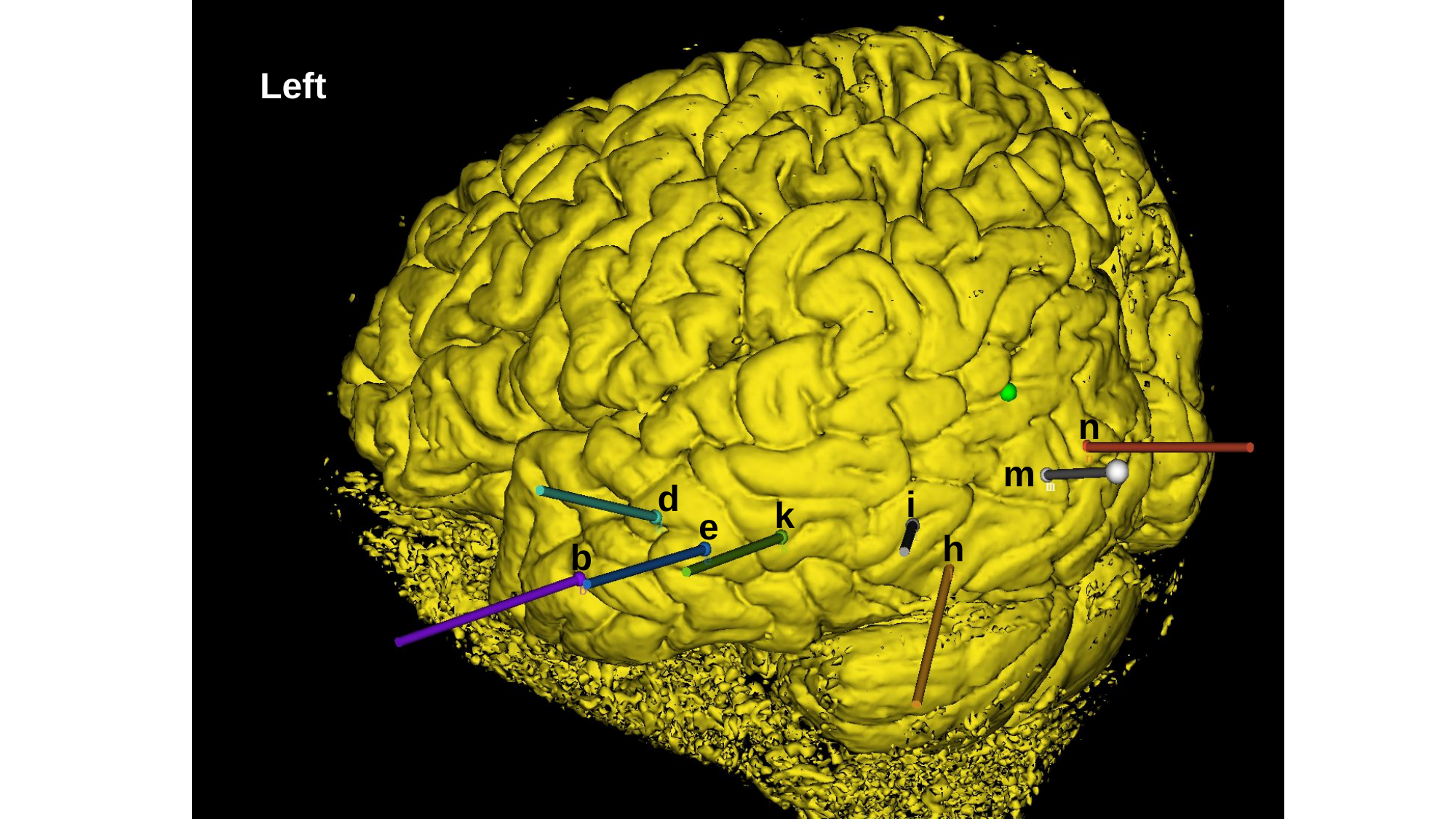

Left
n
m
d
i
k
e
h
b

## Slide 11
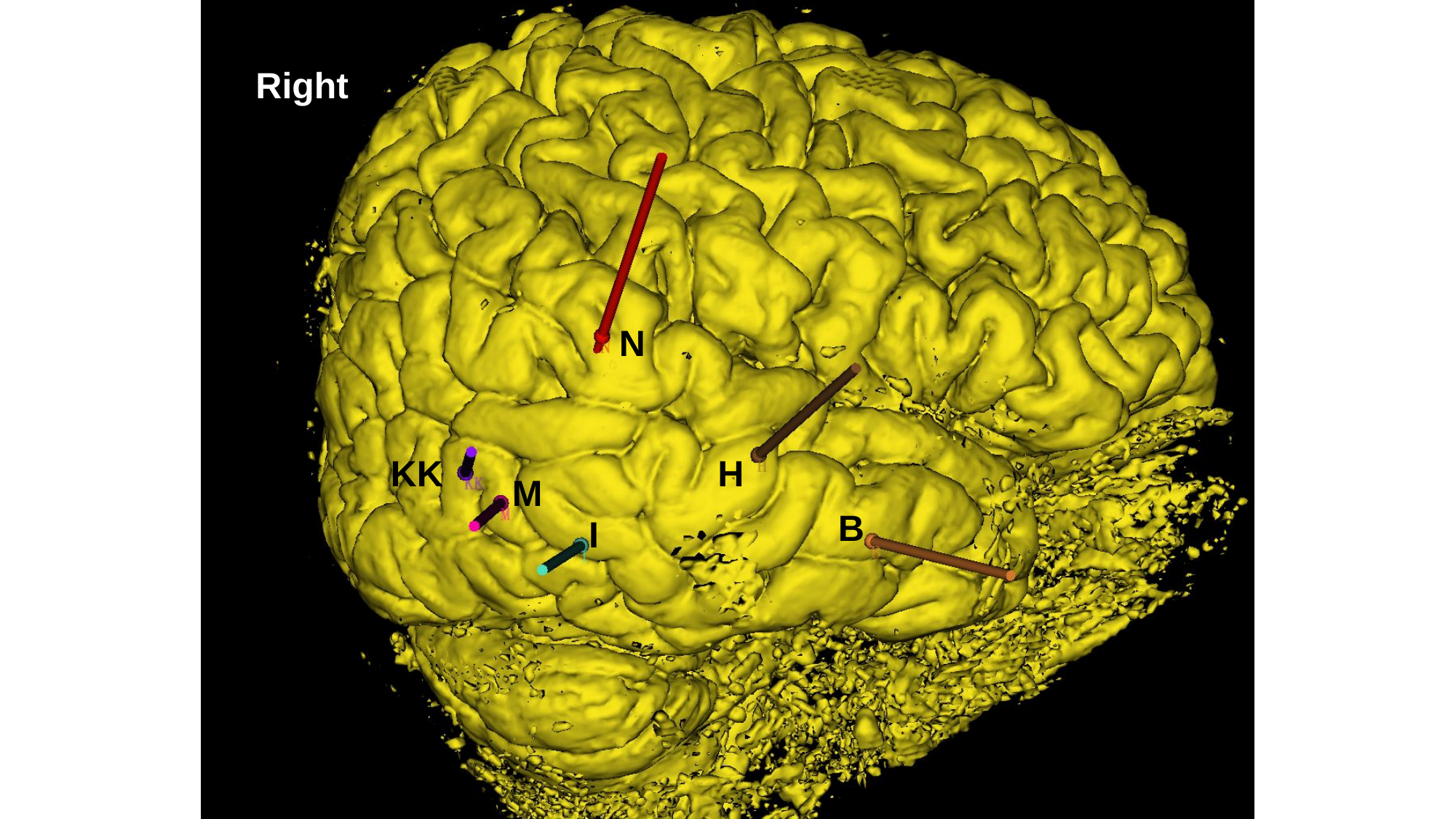

Right
N
KK
H
M
B
I
