# Supplementary material for: RELN gene-related drug-resistant epilepsy with periventricular nodular heterotopia treated with radiofrequency thermocoagulation: a case report
Source: Front Neurol. 2024 Mar 27;15:1366776. doi: 10.3389/fneur.2024.1366776 (PMC11004351; doi:10.3389/fneur.2024.1366776)
Supplement: Supplementary file 5 [file Table_2.DOCX]

| **monopolar coagulation** | **monopolar coagulation** | **monopolar coagulation** | **monopolar coagulation** | **monopolar coagulation** |
| --- | --- | --- | --- | --- |
| m2-m3 | n3-n4 | h1-h2 | i1-i2 | k1-k2 |
| m3-m4 | n4-n5 | h2-h3 | i3-i4 | k2-k3 |
| m4-m5 | n5-n6 | h3-h4 | i4-i5 | k3-k4 |
| m7-m8 | n6-n7 | h4-h5 | i5-i6 | k4-k5 |
| m8-m9 | n12-n13 | h7-h8 |  | k5-k6 |
| m9-m10 |  | h8-h9 |  |  |
|  |  | h10-h11 |  |  |
|  |  | h11-h12 |  |  |
| **monopolar coagulation** | **monopolar coagulation** | **monopolar coagulation** | **bipolar coagulation** | **bipolar coagulation** |
| e3-e4 | d5-d6 | b4-b5 | m3-n5 | b5-d6 |
| e4-e5 | d6-d7 | b5-b6 | m4-n6 | b6-d7 |
|  |  |  | m5-n7 |  |
|  |  |  | m3-h2 |  |
|  |  |  | m4-h3 |  |
|  |  |  | n4-h1 |  |
|  |  |  | n5-h2 |  |

**Coagulation contacts (left electrodes)**

**Coagulation contacts (right electrodes)**

| **monopolar coagulation** | **monopolar coagulation** | **monopolar coagulation** | **monopolar coagulation** | **monopolar coagulation** |
| --- | --- | --- | --- | --- |
| KK2-KK3 | N1-N2 | I6-I7 | H4-H5 | B6-B7 |
| KK3-KK4 | N2-N3 | I7-I8 | H5-H6 | B7-B8 |
| KK4-KK5 | N8-N9 | I8-I9 | H6-H7 |  |
|  | N9-N10 |  | H7-H8 |  |
|  | N10-N11 |  |  |  |
|  | N11-N12 |  |  |  |
|  | N12-N13 |  |  |  |
